# Molecular Classification in Patients With Endometrial Cancer After Fertility-Preserving Treatment: Application of ProMisE Classifier and Combination of Prognostic Evidence

**DOI:** 10.3389/fonc.2022.810631

**Published:** 2022-05-19

**Authors:** Xuting Ran, Tingwenyi Hu, Zhengyu Li

**Affiliations:** ^1^Department of Gynecology and Obstetrics, West China Second University Hospital, Sichuan University, Chengdu, China; ^2^Key Laboratory of Birth Defects and Related Diseases of Women and Children (Sichuan University), Ministry of Education, Chengdu, China

**Keywords:** molecular classification, endometrial cancer, fertility-preserving treatment, ProMisE classifier, progestin

## Abstract

The Proactive Molecular Risk Classifier for Endometrial Cancer (ProMisE) is a molecular classification system that identifies endometrial cancer (EC) into four prognostically distinct subtypes: *POLE*-mutated, mismatch repair deficiency (MMR-D), p53 wild-type (p53wt), and p53 abnormal (p53abn). However, few reports have applied the ProMisE classifier to EC patients who underwent fertility-preserving treatment (FPT) so far. This study evaluated whether the ProMisE classifier predicted in early-stage EC patients after FPT. We first summarized the three reported outcomes of ProMisE applied to EC patients who received FPT. The hormone-treated patients with EC from 2010 to 2020 in our facility were then analyzed. By sequential immunohistochemistry and Sanger sequencing of *POLE* according to the ProMisE system, formalin-fixed paraffin-embedded blocks of patients before treatment were collected and classified into *POLE*-mutated, MMR-D, p53wt, and p53abn subtypes. The primary outcome was a complete response rate after FPT. Thirteen patients were enrolled from our facility, with 3 (3/13) MMR-D, 0 (0/13) POLE, 8 (8/13) p53wt, 1 (1/13) p53abn, and 1 (1/13) failed with DNA amplification. Six (6/8) patients with p53wt, 2 (2/3) patients with MMR-D, and 1 (1/1) patient with p53abn achieved a complete response in 6 months after treatment. The results of our study and the reported outcomes were finally combined. A total of 106 patients who underwent FPT were included. Of these, 23 (21.7%) were classified as MMR-D, 3 (2.8%) as *POLE*-mutated, 3 (2.8%) as p53abn, and 77 (72.6%) as p53wt. There was no significant difference in the complete response rate (P = 0.152) and recurrence rate (P = 0.174) between MMR-D and p53wt subtypes after FPT. Based on current data, we observed no prognostic significance of the ProMisE classifier in EC patients who underwent FPT. Larger prospective studies are needed to elucidate the precise prognostic meaning of this molecular classifier in these cases.

## Introduction

Endometrial cancer (EC) is one of the most common gynecological malignancies worldwide, with approximately 417,000 cases diagnosed in 2020 (1). Almost 7% of new cases occur in women under age 44, and the incidence is increasing due to obesity and other risk factors (2). Although the gold standard treatment for patients with EC is total hysterectomy with bilateral salpingo-oophorectomy, fertility-preserving treatment (FPT) could be considered in FIGO stage IA, lesions confined to the endometrium or superficial myometrium, and grade 1 endometrioid EC patients who desire pregnancy in the future (3). Current FPT options include oral progestin agents (medroxyprogesterone acetate or megestrol acetate) or the levonorgestrel intrauterine system (LNG-IUS), with a 79.4% overall complete response rate in a recent meta-analysis (4).

Although fertility-sparing management for endometrial cancer has increasingly been investigated, the selection of patients suitable for FPT with pathologic examination and magnetic resonance imaging (MRI) still has shortfalls due to a lack of consensus among pathologists and an unreproducible diagnosis of histotype and grade of EC (5). Besides, although patients eligible for FPT are those younger than 40 years with well-differentiated, endometrioid EC clinically limited to the endometrium and no extra-uterine disease, these indications are not restricted in clinical practice. FPT appears to be feasible even in age > 40 years (6), with grade 2 endometrioid adenocarcinoma (7), or with minimal myometrial infiltration (8). In this scenario, a better tool of a molecular classifier is needed to help direct patient management and identify patients for whom conservative management is safe.

The Cancer Genome Atlas (TCGA) endometrial collaborative project discovered four distinct prognostic EC genomic subtypes (9). Since it was costly and complex for clinical application, the Proactive Molecular Risk Classifier for Endometrial Cancer (ProMisE), which is simple and suitable in clinical practice, was developed and stratified EC into 4 subgroups: (i) mismatch repair deficient (MMR-D), showing the loss of one or more mismatch repair protein(s); (ii) DNA polymerase epsilon (POLE), with mutations in the exonuclease domain in exons 9–14, is associated with very favorable outcomes; (iii) p53 abnormal (p53abn) demonstrating aberrant p53 immunohistochemical staining; and (iv) p53 wild type (p53wt) (10). This classifier has been validated and applied to EC patients who underwent standard surgical treatment. However, its application to EC patients who received FPT is relatively novel, and there are only a few studies tested on a cohort of EC patients who were conservatively treated, with differences in outcomes among these studies (11–13). This study further validated whether the ProMisE classifier could predict treatment response in women with endometrial cancer who underwent fertility-sparing treatment.

## Materials and Methods

### Search Strategy, Data Extraction and Quality Assessment

First, we included original studies with primary data reporting the prognostic significance of the Proactive Molecular Risk Classifier for EC in the fertility-sparing management of endometrial cancer. We searched for peer-reviewed studies published before 1 November 2021, in the MEDLINE, Embase, and Google Scholar databases with various combinations of the following keywords: fertility-sparing treatments, progesterone, intrauterine devices, and early endometrial cancer. Studies were selected if the participants were women diagnosed histologically with early-stage EC, the intervention was fertility-sparing therapy, patients were classified according to the ProMisE for molecular subtypes, and a complete response (CR) rate, a partial response (PR) rate, a and relapse rate (RR) were included in the outcomes.

Studies were hand-searched and selected in a 2-stage process. First, the titles and abstracts from the electronic searches were scrutinized by 2 reviewers independently (XR and TH), and full manuscripts of all citations that met the pre-defined selection criteria were obtained. Second, final inclusion or exclusion decisions were made on the examination of the full manuscripts.

Data were extracted independently by 2 reviewers (XR and TH). We extracted data on the study population (number, age, BMI, percentage of molecular subtypes, and follow-up time), and the major outcomes. We assessed study quality using items from the Newcastle–Ottawa Quality Assessment Scale and the Quality Assessment of Diagnostic Accuracy Studies tool (14) (**Supplementary Table 1**).

Two reviewers (XR and TH) independently assessed the risk of bias of each study using the Quality in Prognosis Studies (QUIPS) tool, which contains several domains: study participation, study attrition, prognostic factor measurement, outcome measurement, study confounding, and statistical analysis and reporting. The risk of bias concerns were rated in each domain as “high risk of bias,” “moderate risk of bias,” or “low risk of bias.” The overall risk of bias was considered low if ≤2 domains were rated as having a moderate risk of bias and all others were rated as having a low risk of bias. The overall risk of bias was considered moderate if >2 domains were rated as having a moderate risk of bias and all others were rated as having a low risk of bias. The overall risk of bias was considered high if ≥1 domain was rated a high risk of bias, irrespective of all other domains (15). Consensus was reached after classification by individual researchers (**Supplementary Table 2**).

### Study Population

We retrospectively reviewed the data of young patients with endometrial cancer who had received FPT at the West China Second University Hospital, Sichuan University, during 2010–2020. The study was approved by the ethics committee of West China Second University Hospital, Sichuan University. The inclusion criteria were as follows (1): aged 18–40 years (2), clinically presumed International Federation of Gynecology and Obstetrics (FIGO) stage IA (3), pathologically diagnosed grade 1, endometrioid adenocarcinoma, and (4) no contraindication for progestin treatment. Exclusion criteria include inadequate quality of tumor tissues, unclear medication history, or no tissue available at our institution. The primary outcome was the complete response rate after FPT for each molecular group. A complete response (CR) was defined as no evidence of residual EC or atypical hyperplasia (AH) at follow-up endometrial sampling, diagnosed by hysteroscopic biopsy. Time until CR was measured from the treatment start date. The other pathologic responses to progestin treatment were defined as follows: Partial regression (PR) was defined as the presence of atypical hyperplasia (AH) during follow-up endometrial sampling by hysteroscopic biopsy. Disease persistence was defined as no evidence of disease regression was observed within 6 months. Disease progression is a lesion of higher grade or clinically progressive disease, including myometrial invasion, extrauterine disease, or lymph node metastasis. Recurrence was defined as the presence of EC or AH during follow-up after an endometrial sample indicated treatment response. Time to recurrence was defined as from the date of the complete response. Patient follow-up data were gathered until the end of 2020.

The evaluation of response before achieving CR was performed using dilation and curettage (D&C) or hysteroscopic biopsy every 3 months during the initial treatment for 2 years, then 6-monthly. Unless there was any evidence of progression in the follow-up endometrial biopsies and/or imaging studies, patients continued to receive initial treatment until they achieved CR.

### Application of the ProMisE Classifier to Endometrial Biopsy Specimens

The FFPE biopsies of patients were evaluated according to the ProMisE algorithm based on IHC for MMR proteins, sequencing for the presence of POLE exonuclease domain mutations, and IHC for p53 (5, 12, 16). Tumors were categorized into one of the four ProMisE molecular subgroups: POLE-mutated, mismatch repair deficient (MMR-D), p53 wild-type (p53wt), and p53-abnormal (p53abn).

#### IHC for MMR and P53 Proteins

In the first step of the algorithm, a representative FFPE block was evaluated for the expression of the MMR proteins, namely, MLH1, MSH2, MSH6, and PMS2 by IHC. Sections were cut at 4 mm thickness and de-waxed in xylene and ethanol before rehydration. Researchers then blocked endogenous peroxidase activity by incubating sections in a 3% H2O2 solution in methanol at room temperature for 10 min. After antigen retrieval with citric acid (pH 6.0) at 121°C for 15 min, the sections were blocked with 10% normal goat serum and incubated overnight at 4°C with four antibodies: anti-hMLH1 (1:1,000; ProteinTech Group, Chicago, IL); anti-hMSH2 (1:100; ProteinTech Group, Chicago, IL); anti-hMSH6 (1:100; ProteinTech Group, Chicago, IL); and anti-hPMS2 (1:1,000; ProteinTech Group, Chicago, IL). After washing with PBS, the sections were incubated with biotinylated rabbit anti-goat immunoglobulin antibody (ZSGB-Bio Ltd., Beijing, China), and the slides were visualized by staining with diaminobenzidine (Dako Ltd., Glostrup, Denmark) and counterstained with hematoxylin. Protein expression in appendix tissue served as an internal positive control. For p53 immunostaining, the slides were incubated with p53 primary antibody (1:100; ProteinTech Group, Chicago, IL). Slides of high-grade serous ovarian cancer were used as positive controls.

We performed image acquisition (BA400Digital; MOTIC China Group Co., Ltd.) and quantitative analysis (Halo 101-WL-HALO-1; Indica Labs, USA) of the slides. The tumor was classified into the MMR-D subtype if IHC demonstrated a loss of MMR protein nuclear expression. Immunostaining for p53 was considered abnormal when there was no staining of tumor cell nuclei or strong and diffuse staining (absent p53 protein or ab-errant increased protein accumulation, respectively), while intermediate levels of expression were considered wild-type.

#### DNA Extraction, Targeted Sequencing for POLE Mutation

Genomic DNA (gDNA) was extracted from FFPE using the Ezup Column Animal Genomic DNA Purification Kit (Sangon Biotech (Shanghai) Co., Ltd.). gDNA was amplified with the forward and reverse primers. After purified PCR products were detected by agarose gel electrophoresis (150 V, 100 mA, 10–20 min), DNA was extracted from the agarose gel by a SanPrep Column DNA Gel Extraction Kit (Sangon Biotech (Shanghai) Co., Ltd.), and then Sanger-sequenced with the BigDye terminator v1.1 sequencing kit and a 3730xl automated sequencer (Applied Biosystems, Foster City, CA, USA). The results were analyzed by Variant reporter software version 2.1 (Applied Biosystems).

### Statistical Analysis

Data were collected retrospectively through chart review. Summary statistics are provided. Normally distributed continuous variables (age and body mass index (BMI)) were compared using Student’s t-test; nonparametric continuous factors were compared using the Wilcoxon rank-sum test and proportions were compared using a chi-square test. Analyses were conducted using SPSS 22.0 (IBM Corp., Armonk, NY, USA).

## Results

### Literature Review of the Three Previous Studies

After literature searching, we found three studies that tested the PromisE classifier on EC patients who received FPT so far (11–13). The characteristics and outcome measures of each study are summarized in **Table 1**. A total of 94 EC patients who underwent hormone therapy were classified by ProMisE in 3 studies and distributed as follows: 20 (21.3%) MMR-D, 3 (3.2%) POLE, 2 (2.1%) p53abn, and 69 (73.4%) p53wt. In the study by Falcone et al. (12), three cases had more than one molecular feature: MMR-D + p53abn (n = 1); MMR-D + POLE-mutated subtypes (n = 2). The rest of all cases demonstrated one molecular feature.

**Table 1:** Molecular characterization of patients with endometrial cancer underwent FPT and outcomes.

Study	Characteristics	MMR-D	*POLE-*Mutated	p53abn	p53wt	*P*^1^	Total
Chung et al. (11)	**Number**	**9 (15.8)**	**2 (3.5)**	**1 (1.8)**	**45 (78.9)**	**–**	**57**
	Age at diagnosis (years)	33 (26–40)	27, 34	33	31 (19–45)	0.382	33 (19–45)
	BMI (kg/m^2^)	24.6 (18.8–41.3)	40.5, 20.2	20.0	26.8 (17.8–39.9)	0.265	25.7 (17.8–41.3)
	CR rate at 6 months	1 (11.1)	1 (1/2)	1 (1/1)	24 (53.3)	0.010	27 (47.4)
	Best overall response of CR/PR rate	4 (44.4)	1 (1/2)	1 (1/1)	37 (82.2)	0.018	43 (75.4)
	Recurrence rate after CR	1 (25.0)	1 (1/2)	1 (1/1)	16 (43.2)	0.629	19 (44.2)
	Hysterectomy	4 (44.4)	2 (2/2)	0 (0/1)	22 (48.9)	1.000	28 (49.1)
Falcone et al. (12)	**Number**	**7 (46.7)**	**1 (6.7)**	**0 (0)**	**7 (46.7)**		**15**
	Age at diagnosis (years)	38 (28–39)	36	–	37 (25–40)	–	37 (25–40)
	BMI (kg/m^2^)	29.0 (24.3–53.5)	38.3	*–*	24.2 (22.7–33.1)	–	26.3 (22.7–53.3)
	CR rate at 6 months	5 (5/7)	1 (1/1)	*–*	7 (7/7)	–	13 (13/15)
	Best overall response of CR/PR rate	5 (5/7)	1 (1/1)	*–*	7 (7/7)	–	13 (13/15)
	Recurrence rate after CR	1 (1/7)	0 (0/1)	*–*	2 (2/7)	–	3 (3/15)
	Hysterectomy	2 (2/7)	0 (0/1)	*–*	2 (2/7)	–	4 (4/15)
Puechl et al. (13)	**Number**	**4 (18.2)**	**0 (0)**	**1 (6.7)**	**17 (77.3)**	–	**22**
	CR rate at 6 months	3 (3/4)	–	0 (0/1)	13 (76.5)	–	–
	Best overall response of CR/PR rate	3 (3/4)	–	0 (0/1)	13 (76.5)	–	16 (72.7)
	Progression or required definitive treatment	1 (1/4)	–	1 (1/1)	4 (23.5)	–	6 (27.3)
	Hysterectomy	1 (1/4)	–	1 (1/1)	4 (23.5)	–	6 (100.0)
Summary of the 3 studies	**Number**	**20 (21.3)**	**3 (3.2)**	**2 (2.1)**	**69 (73.4)**	–	**94**
	CR rate at 6 months	9 (45.0)	2 (2/3)	1 (1/2)	44 (63.8)	0.195	56 (59.6)
	Best overall response of CR/PR rate	12 (60.0)	2 (2/3)	1 (1/2)	57 (82.6)	0.040	72 (76.6)
	Recurrence rate after CR	3 (15.0)	1 (1/3)	2 (2/2)	22 (31.9)	0.168	28 (29.8)
	Hysterectomy	7 (35.0)	2 (2/3)	1 (1/2)	28 (40.6)	0.796	38 (40.4)

Data are presented as median (range) or n (%). ^1^Patients with POLE-mutated or p53abn were excluded.

MMR-D, mismatch repair deficient; abn, abnormal; wt, wild type; BMI, body mass index; CR, complete regression; PR, partial response; -, not available.

The oncologic outcomes of the three studies are presented. Chung et al. reported the following CR rates at 6 months of the four ProMisE subtypes: 1 (11.1%) MMR-D, 1 (1/2) POLE, 1 (1/1) p53abn, and 24 (53.3%) p53wt; best overall response of CR/PR rate: 4 (44.4%) MMR-D and 37 (82.2%) p53wt; recurrence rate after CR: 1 (25.0%) MMR-D, 1 (1/2) POLE, 1 (1/1) p53abn, and 16 (43.2%) p53wt. Falcone et al. stated the CR rate at 6 months as follows: 5 (5/7) MMR-D, 1 (1/1) POLE, and 7 (7/7) p53wt; recurrence rate after CR: 1 (1/7) MMR-D and 2 (2/7) p53wt. Puechl et al. reported a CR rate at 6 months: 3 (3/4) MMR-D and 13 (76.5%) p53wt, with 1 (1/4) MMR-D, 1 (1/1) p53abn, and 4 (23.5) p53wt having progression or requiring definitive treatment. After combining the results of three studies, patients with MMR-D showed a lower overall response rate of CR/PR rate than those with the p53wt subtype (60.0% vs 82.6%, P = 0.040), which was consistent with the result of Chung et al. (11). The combined CR rate after 6 months of hormone therapy in the MMR-D group had no significant difference compared with p53wt subgroup, however, Chung et al. (11) reported a lower rate in MMR-D patients. Puechl et al. (13) reported that 1/4 of EC patients with MMR-D and 4/17 with p53wt developed progression or underwent definitive surgery after levonorgestrel intrauterine system (LNG-IUS) treatment. They also included 37 endometrial intraepithelial neoplasia (EIN) patients. After adding them to EC patients (n = 59), p53abn tumors exhibited the shortest time to progression or definitive therapy. Due to the small sample size, Falcone et al. (12) did not conclude that there were significant differences in outcomes among subtypes.

From combined studies, we found that, similar to the result of Chung et al. (11), the MMR-D subtype was associated with a worse overall response rate compared to the p53wt subtype in women with EC who underwent FPT. However, larger samples changed the comparison outcomes of the CR rate at 6 months after treatment between MMR-D and p53wt subtypes. Therefore, studies are needed to further test whether the MMR-D subtype could predict response in women with endometrial cancer treated conservatively. Besides, owing to the limited sample size, the POLE-mutated and p53abn subtypes in predicting hormone response also need to be further explored.

### Results of the Current Study

**Figure 1:**
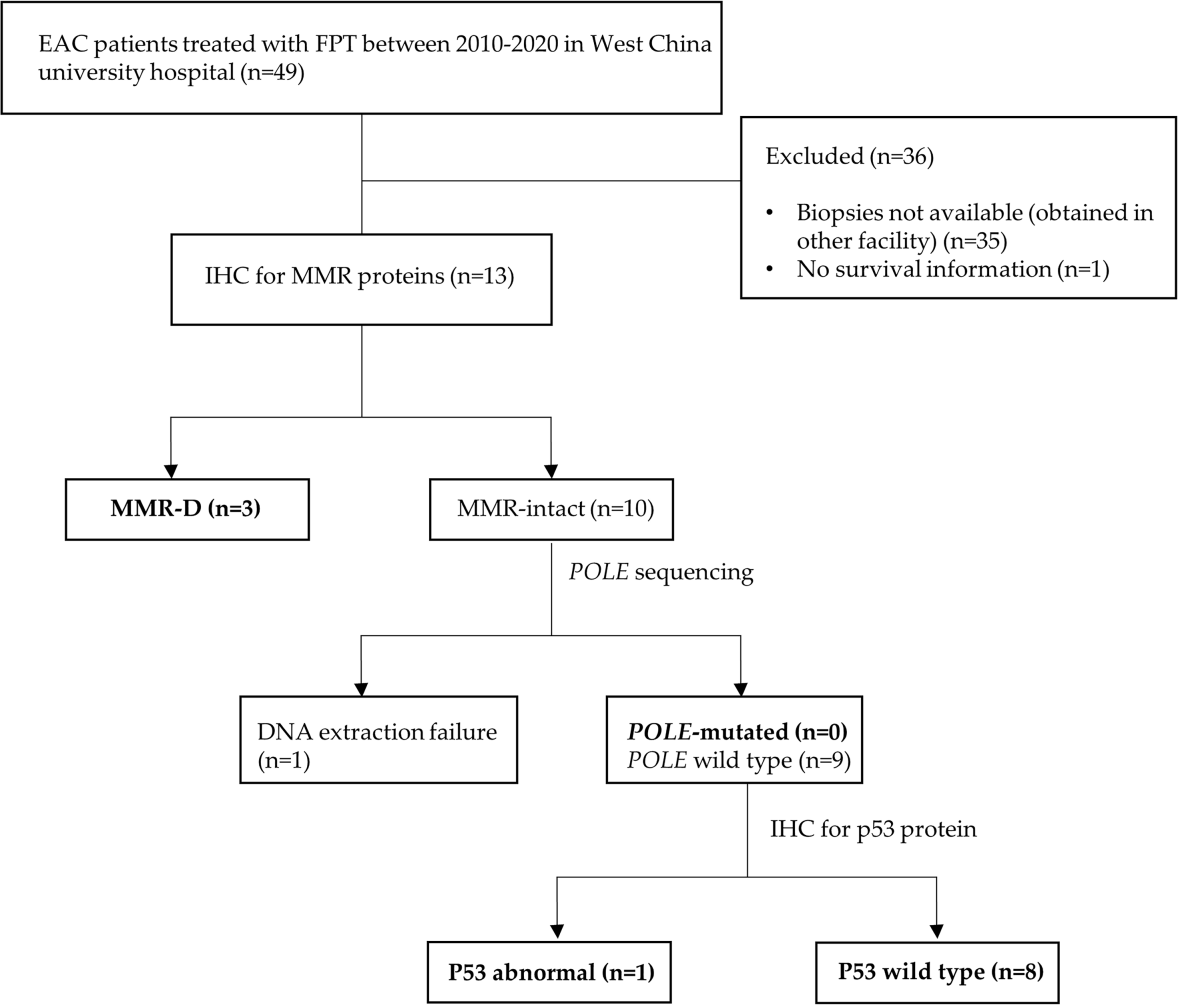
Flow chart of patient selection and ProMise algorithm apllication.

Owing to the limited samples and changed outcomes after the combination of previous studies, we performed further exploration to test the prognostic ability of the ProMisE classifier in EC patients who underwent FPT. Thirteen patients with Grade 1 endometrioid adenocarcinoma who underwent FPT; pretreatment formalin-fixed, paraffin-embedded (FFPE) tissues in our institution were included. The study flowchart is shown in **Figure 1**. A total of 49 Grade 1 endometrioid adenocarcinoma (EAC) patients who underwent FPT between 2010 and 2020 in the West China University Hospital were first identified; 35 patients had their pre-treatment biopsies obtained in other hospitals, and 1 patient lost survival information. Finally, 13 patients were enrolled in the ProMisE algorithm application.

Immunohistochemistry (IHC) for MMR proteins was performed in the first step. Three cases exhibited a loss of MMR proteins and were categorized as MMR-D subtype. MMR-intact cases were then evaluated for POLE mutations by Sanger sequencing. Nine cases showed POLE wild-type and one case failed DNA amplification. Then, POLE wild-type cases were subjected to IHC for p53, one case showed p53 overexpression and was classified as p53abn subtype. In summary, the molecular classification of the 13 cases presented the following ProMisE subtypes: 3 (3/13) MMR-D, 0 (0/13) POLE, 8 (8/13) p53wt, 1 (1/13) p53abn, and 1 (1/13) failed with DNA amplification. No cases showed more than one molecular feature. The detailed molecular features of all patients are summarized in **Table 2**.

**Table 2:** Molecular classification by ProMisE algorithm of all the patients.

Patients (No.)	Age at diagnosis (years)	ProMisE subtype	MMR IHC abnormal	*POLE* mutation	p53 IHC
1	31	p53wt	–	–	p53wt
2	35	MMR-D	MSH6	–	p53wt
3	23	p53wt	–	–	p53wt
4	29	p53wt	–	–	p53wt
5	28	p53wt	–	–	p53wt
6	33	p53wt	–	–	p53wt
7	26	–	–	DNA amplification failure	p53wt
8	34	p53abn	–	–	p53abn
9	29	p53wt	–	–	p53wt
10	23	p53wt	–	–	p53wt
11	29	p53wt	–	–	p53wt
12	31	MMR-D	MSH6	–	p53wt
13	37	MMR-D	MSH2; MSH6	–	p53wt

IHC, immunohistochemistry; MMR, mismatch repair; MSH6, MutS homolog 6; MSH2, MutS homolog 2; MMR-D, mismatch repair deficient.

The clinical–pathological characteristics, treatment, oncologic, and pregnancy outcomes of the patients are detailed in **Table 3**. The mean age of the cohort was 29.8 ± 4.3 years and the mean body mass index (BMI) was 25.4 ± 7.1 (kg/m^2^). All patients had an initial diagnosis of Grade 1 endometrioid adenocarcinoma (EAC). Four of thirteen cases were diagnosed *via* hysteroscopy, and 9/13 *via* dilation and curettage (D&C). Patients received treatment, namely, megestrol acetate (MA), medroxyprogesterone acetate (MPA), goserelin acetate, and levonorgestrel. The average time of the treatment was 7.7 months, ranging from 3 to 24 months. Ten of thirteen patients had a complete response after hormone treatment at 6 months. Patient No. 3 was found with disease progression and an ovarian mass after 107 months of complete response and was treated by definitive surgery, showing a stage IA G2 endometrioid ovarian cancer (OC) and synchronous asymptomatic endometrioid G2 EC.

**Table 3:** Demographics, clinical–pathological characteristics, treatment, oncologic outcomes of the EC patients who underwent FPT.

Patients (No.)	Age at diagnosis (years)	BMI (kg/m^2^)	Pre-diagnosis GxPx	Pathology	Diagnostic method	Treatment, mg/day		Treatment duration, months
1	31	21.9	G0P0	G1 EAC	D&C	MA, 160 + MPA, 250		3 +3
2	35	20.4	G2P1	G1 EAC	Hysteroscopy	MA, 160		3
3	23	20.7	G0P0	G1 EAC	D&C	MA, 160		6
4	29	18.4	G1P0	G1 EAC	D&C	MA, 160		3
5	28	30.8	G0P0	G1 EAC	Hysteroscopy	MA, 160 + LNG-IUS		6 + 5
6	33	37.6	G0P0	G1 EAC	D&C	MA, 160 + LNG-IUS		6
7	26	26.6	G0P0	G1 EAC	Hysteroscopy	MPA, 250 + LNG-IUS		10
8	34	39.4	G2P0	G1 EAC	Hysteroscopy	MPA, 500		24
9	29	30.7	G0P0	G1 EAC	Hysteroscopy	LNG-IUS		12
10	23	24.8	G0P0	G1 EAC	Hysteroscopy	LNG-IUS		11
11	29	18.6	G0P0	G1 EAC	Hysteroscopy	MA, 160		3
12	31	20.4	G0P0	G1 EAC	Hysteroscopy	MPA, 250 + LNG-IUS		4
13	37	20.3	G0P0	G1 EAC	Hysteroscopy	MA, 160 + GnRH-A (i.m)		1month + 4 times
Patients (No.)	Follow-up duration, months	Oncologic outcome at 6 months	Final diagnosis	Recurrence (months)	Second cancer (months)	Hysterectomy	Current status	GxPx at the end of the treatment
1	138	CR	Normal	–	–	No	NED	G0P0
2	120	CR	Normal	–	–	No	NED	G2P1
3	123	CR	Normal	107	Ovarian cancer (107)	Yes	AWD	G0P0
4	84	CR	Normal	–	–	Yes	NED	G1P0
5	60	Progression^1^	EAC	–	–	Yes	NED	G0P0
6	48	CR	Normal	–	–	No	NED	G0P0
7	46	CR	Normal	–	–	No	NED	G0P0
8	27	PR	Normal	–	–	No	NED	G2P0
9	15	CR	Normal	–	–	No	NED	G0P0
10	11	CR	Normal	–	–	No	NED	G0P0
11	12	Persistent	EAC	–	–	Yes	NED	G0P0
12	15	CR	Normal	–	–	No	NED	G0P0
13	13	Persistent	EAC	–	–	Yes	NED	G0P0

^1^Definitive surgery at 5 months.

BMI, body mass index; G, gravida; P, para; G1, Grade 1; D&C, dilation and curettage; EAC, endometrioid adenocarcinoma; MA, megestrol acetate; MPA, medroxyprogesterone acetate; LNG-IUS, Levonorgestrel-releasing intrauterine system; GnRH-A, Gonadotropin-releasing hormone agonist; i.m, intramuscular injection; CR, complete response; AWD, alive with disease; NED, no evidence of disease.

Three patients had disease progression and persistent disease, respectively. Patient No. 5 had p53wt mutation-developed disease progression after 6 months of MA plus 5 months of LNG-IUS therapy. The final diagnosis showed stage IA G1 endometrioid EC. Patient No. 11 with p53wt mutation kept persistent after 3 months of MA treatment, then underwent definitive surgery immediately. Patient No. 13 with MMR-D underwent genetic testing and did not suggest Lynch syndrome. MRI of the patient showed minimal myometrial infiltration and it was not recommended to continue FPT after 1 month of MA treatment and 4 GnRH-A injections. Overall, 12 patients were alive and had no evidence of disease; 1 patient was alive with ovarian cancer at the end of the follow-up.

Finally, we combined our results with the three previous studies. The molecular characterization and outcomes are detailed in **Table 4**. A total of 106 EC patients who underwent hormone therapy were included after the combination of the 4 studies. p53wt was still the most common subtype and was observed in 77 cases (72.6%). MMR-D was found in 23 cases (21.7%) and was presented as the second most common subtype. Patients were classified as the following subtypes: 23 (21.7%) MMR-D, 3 (2.8%) POLE-mutated, 3 (2.8%) p53abn, and 77 (72.6%) p53wt. We compared the outcomes of the MMR-D subtype and the p53wt subtype, and we found there was no significant difference in the CR rate after treatment at 6 months between the two groups (47.8% vs. 64.9%, P = 0.152) and overall response of CR/PR rate (60.9% vs. 80.5%, P = 0.092). Recurrence rates were 13.0 and 29.9% in the MMR-D and p53wt subtypes, respectively, which also showed no significant difference (P = 0.174). Of 43 patients with treatment failure or progression who underwent hysterectomy, there was no significant difference between the two groups either.

**Table 4:** Molecular characterization and outcomes of the 4 studies.

Study	Characteristics	MMR-D	*POLE-*Mutated	p53abn	p53wt	*P*^1^	Total
Summary of the 4 studies	Number	23 (21.7)	3 (2.8)	3 (2.8)	77 (72.6)	–	106
	CR rate at 6 months	11 (47.8)	2 (2/3)	2 (2/3)	50 (64.9)	0.152	64 (60.4)
	Best overall response of CR/PR rate	14 (60.9)	2 (2/3)	2 (2/3)	62 (80.5)	0.092	80 (75.6)
	Recurrence rate after CR/Progression	3 (13.0)	1 (1/3)	2 (2/3)	23 (29.9)	0.174	29 (27.4)
	Hysterectomy	8 (34.8)	2 (2/3)	1 (1/3)	32 (41.6)	0.633	43 (40.6)

^1^Patients with POLE-mutated (n = 3) or p53abn (n = 3) were excluded.

## Discussion

This study evaluated the prognostic ability of the ProMisE classifier in early-stage EC patients after FPT. We found no significant difference in the CR rate (P = 0.152) between the MMR-D and p53wt subtypes after FPT. The other oncologic outcomes, including overall response, CR/PR rate, and recurrence rate, also showed no significant difference. Based on current results, we observed no prognostic significance for the ProMisE classifier in EC patients who underwent FPT.

Due to the inadequate ability to refine prognostication or assess treatment efficacy in the last decade, the traditional systems of histo-morphological classification and risk group stratification of EC have been challenged by molecular-based classification. The Cancer Genome Atlas (TCGA) endometrial collaborative project discovered four distinct prognostic EC subtypes based on genomic abnormalities, identifying four molecularly defined prognostic subgroups (10). Later, the analogous but simplified ProMisE classifier was developed and validated, showing high concordance between diagnostic and hysterectomy specimens (5, 17). A few previous studies have applied the ProMisE classifier to EC patients who were conservatively treated, and our study further tested the ProMisE molecular classification system on EC patients who underwent FPT. Our results showed a two-type molecular heterogeneity (MMR-D and p53wt subtypes) within a group of G1 EACs and relatively homogeneous good survival outcomes at present.

We found 3/13 cases of MMR-D at IHC analysis. Patient No. 13 with MMR-D did not suggest Lynch syndrome and had a hysterectomy due to persistent disease. The other two MMR-D patients had a good response to hormone therapy, and they did not undergo LS testing. Similar to the small sample study by Falcone et al. (12), they found about 50% (7/15) MMR defects at IHC analysis and clinical outcome, with 4 of 7 mutated patients showing EC persistence/progression or metachronous Lynch syndrome-associated tumors. The different distributions and outcomes were likely due to the study populations.

We finally combined the outcomes of four studies with the same purpose. The combined results showed four ProMisE subtype distributions as follows: 77 (72.6%) p53wt, 23 (21.7%) MMR-D, 3 (2.8%) POLE-mutated, and 3 (2.8%) p53abn. There were no significant differences in CR rate and recurrence rate between MMR-D and p53wt subtypes, which broke the hypotheses based on validated ProMisE in large cohorts of EC patients and indicated that MMR-D could predict good or poor responses to FPT in EC patients. Our results were different from the pre-combined study and opinions (11, 18–20). The reason for this difference may be attributed to the study population due to the lack of mechanism evidence between MMR deficiency and hormone therapy in EC patients. From previous clinical studies, we also found a controversial relationship between MMR-D and hormone-treated outcomes of EC. Britton et al. (18) first applied the ProMisE classifier to a large cohort of young EC women (<50 years of age) after ProMisE was validated in the non-age stratified cohorts (17) and reported that MMR-D is associated with poorer outcomes compared to p53wt, including overall, disease-specific, and progression-free survival. However, several studies hold different opinions about whether MMR-D can be a predictive biomarker for hormone therapy in EC women. Zakhour et al. (21) found 5/84 EAC patients with MMR-D who underwent progestin therapy and none of the MMR-D patients responded to progestin. Chung et al. (11) found MMR-D had poor outcomes after hormone therapy in early-stage EC. Gallos et al. (22) found no associations between MLH1 protein expression and regression/relapse of women with endometrial hyperplasia treated with LNG-IUS. A hypothesis indicated that MMR-deficient may reduce the function of progesterone receptor (23), however, its reaction to progesterone therapy remained unclear (24, 25). In this regard, further validation is needed to assess the relationship between MMR-D and progestin response in EC patients who received FPT.

Our results showed rare POLE mutated and p53abn subtypes and a much higher proportion of p53wt and MMR-D subtypes than the EC cohort <50 yo at diagnosis (18). A possible reason for this difference may be the loss of POLE patients during the selection of large volume tumor blocks for DNA extraction or population characteristics.

Other factors that have been studied to predict the efficacy of fertility sparing treatment of EC. Travaglino et al. (26) assessed the predictive significance of PTEN and found it seemed not to be useful as a predictive marker of response to conservative treatment of EC, suggesting that PTEN is not applicable as a stand-alone predictive marker. Raffone et al. (27) reported that mismatch repair protein deficiency appears as a highly specific predictor of recurrence of EEC after initial regression. Clinical factors, such as longer menstrual cycles and infrequent menstrual bleeding, also appeared as independent predictive factors for conservative treatment failure in EEC (28). Weak stromal expression of isoform B of the progesterone receptor (PRB) was also found as a highly sensitive predictive marker of both no response and recurrence of EEC conservatively treated (29). Besides, many of the non-coding RNAs (ncRNAs) also reported a prognosis prediction function and remarkable importance in defining the therapeutic and surveillance path of EC patients, such as lncRNA and sncRNA (30). Both lncRNA and sncRNA functionally interact with EC diagnostic and prognostic genes and may be a valuable alternative marker for risk evaluation to aid patient-tailored treatment and improve the outcome of patients with EC (31). Further prospective studies, suggest that all of these factors, combined with the ProMisE classifiers, might represent valuable biomarkers to improve risk stratification for EC patients who underwent fertility sparing treatment.

This study combined the eligible outcomes of the FPT response with the implementation of ProMisE in EC patients. Based on current data, we observed no prognostic information from the classifier for conservatively treated patients. However, this study is limited by the lack of assessment of outcomes for POLE-mt and p53abn patients due to the limited sample size, retrospective nature, and heterogeneity in classification methods across a combination of several studies. Future evaluation is warranted to determine whether molecular classification predicts outcomes for patients considering hormone therapy for endometrial cancer.

## Data Availability Statement

The original contributions presented in the study are included in the article/**Supplementary Material**. Further inquiries can be directed to the corresponding author.

## Ethics Statement

The studies involving human participants were reviewed and approved by the ethics committee of West China Second University Hospital, Sichuan University. The ethics committee waived the requirement of written informed consent for participation.

## Author Contributions

Methodology, formal analysis, investigation, writing—original draft preparation, XR. Methodology, formal analysis, investigation, TH. Conceptualization, formal analysis, investigation, resources, data curation, writing—review and editing, supervision, project administration, funding acquisition, ZL. All authors listed have made a substantial, direct, and intellectual contribution to the work and approved it for publication.

## Funding

This research was funded by the Department of Science and Technology of Sichuan Province, grant number 2019YJ0072.

## Conflict of Interest

The authors declare that the research was conducted in the absence of any commercial or financial relationships that could be construed as a potential conflict of interest.

## Publisher’s Note

All claims expressed in this article are solely those of the authors and do not necessarily represent those of their affiliated organizations, or those of the publisher, the editors and the reviewers. Any product that may be evaluated in this article, or claim that may be made by its manufacturer, is not guaranteed or endorsed by the publisher.
