# Supplementary material for: Molecular Classification in Patients With Endometrial Cancer After Fertility-Preserving Treatment: Application of ProMisE Classifier and Combination of Prognostic Evidence
Source: Front Oncol. 2022 May 19;12:810631. doi: 10.3389/fonc.2022.810631 (PMC9160735; doi:10.3389/fonc.2022.810631)
Supplement: Supplementary file 1 [file DataSheet_1.docx]

Supplementary Material

**p53**

**PMS2**


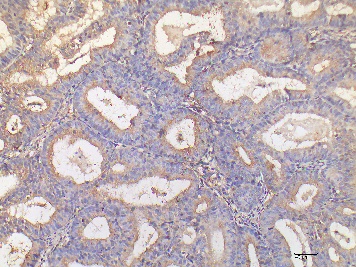

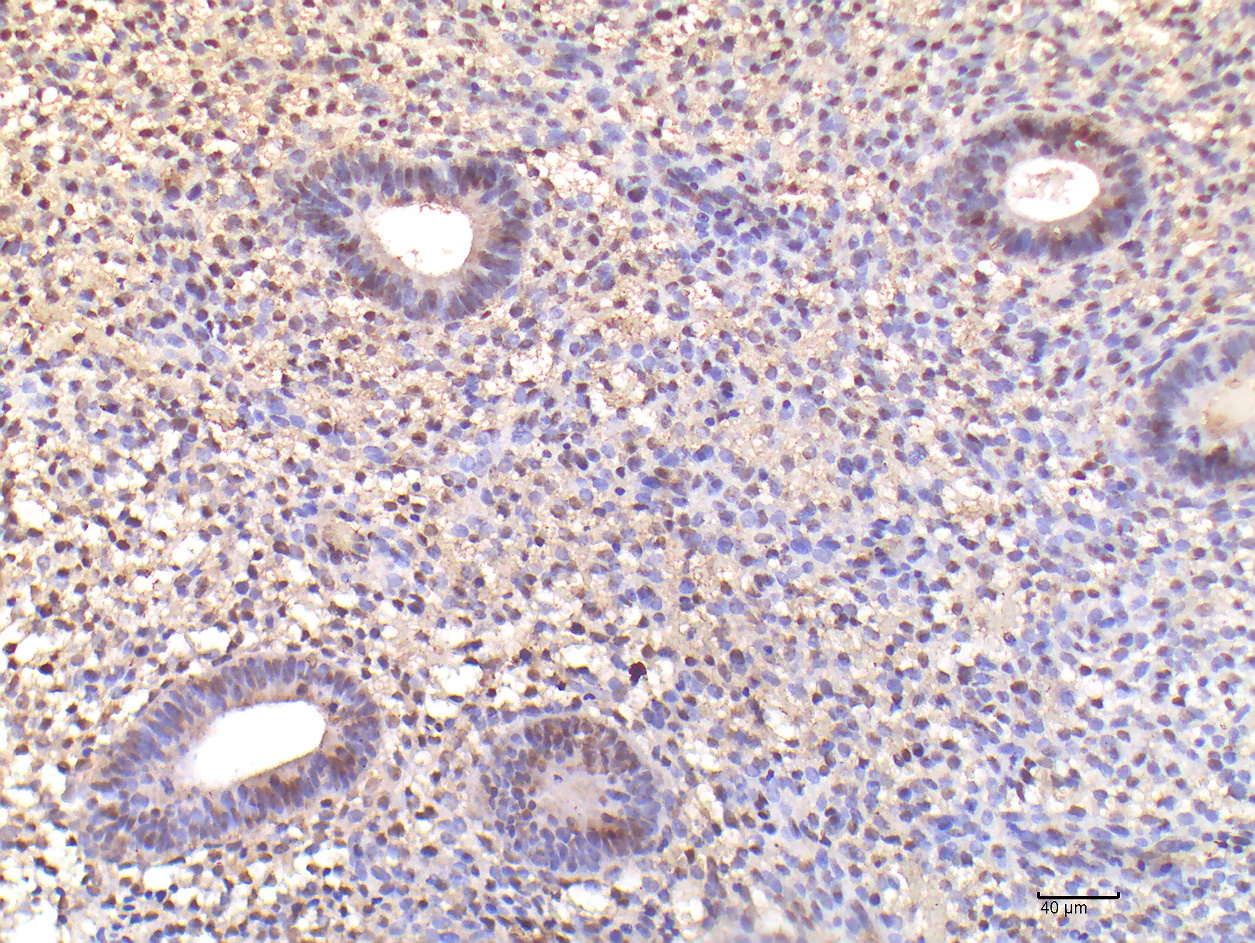

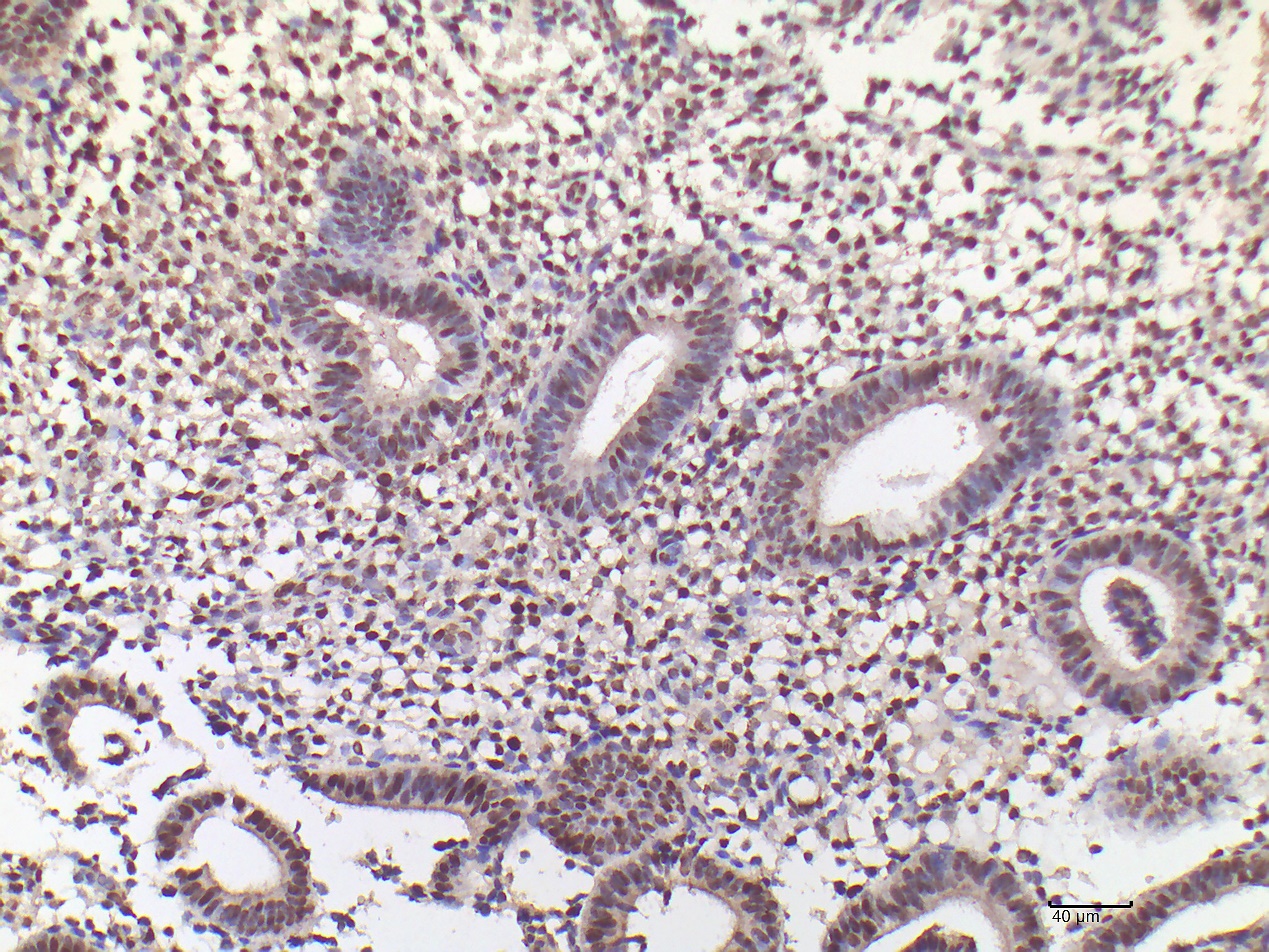

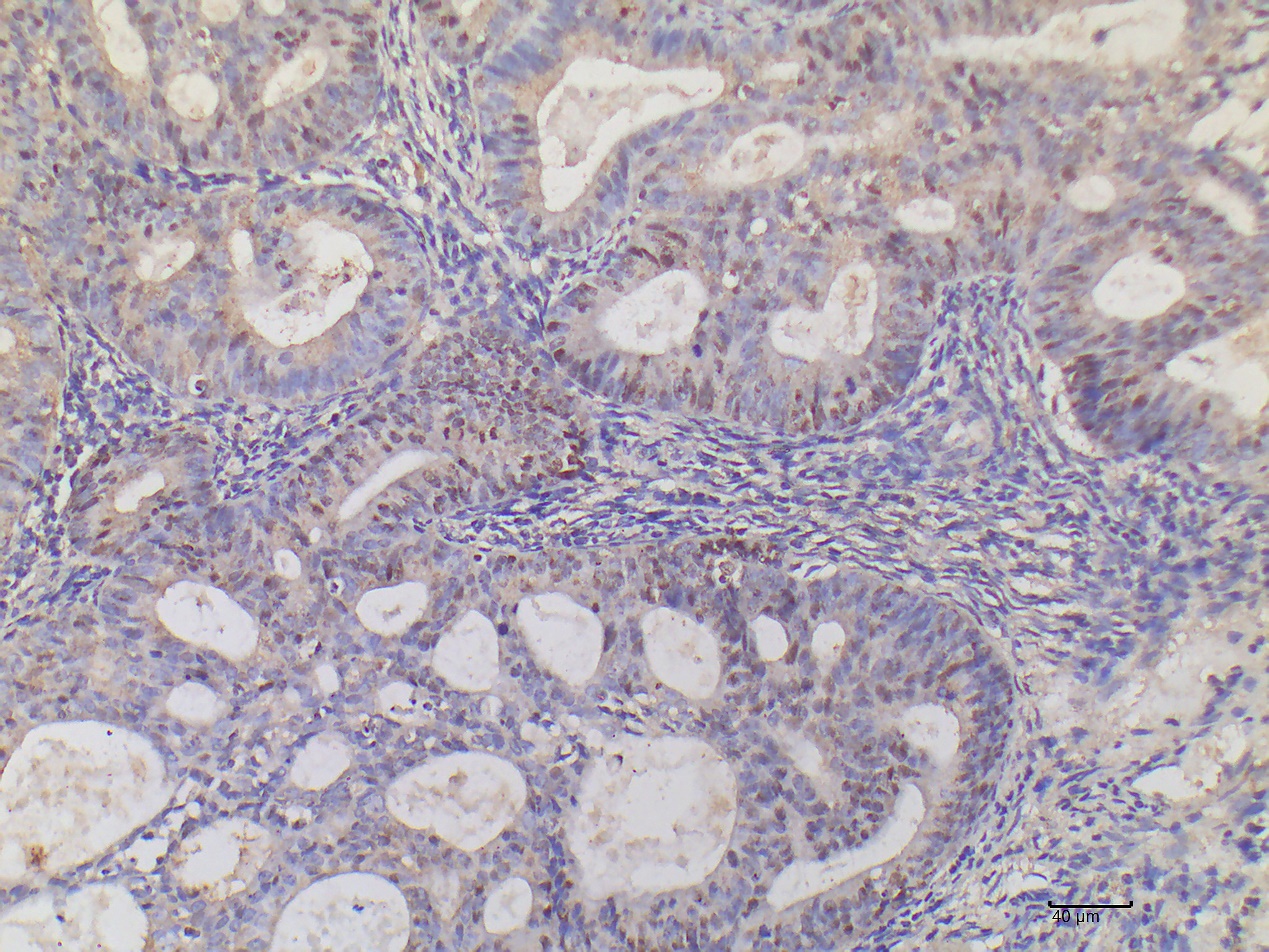

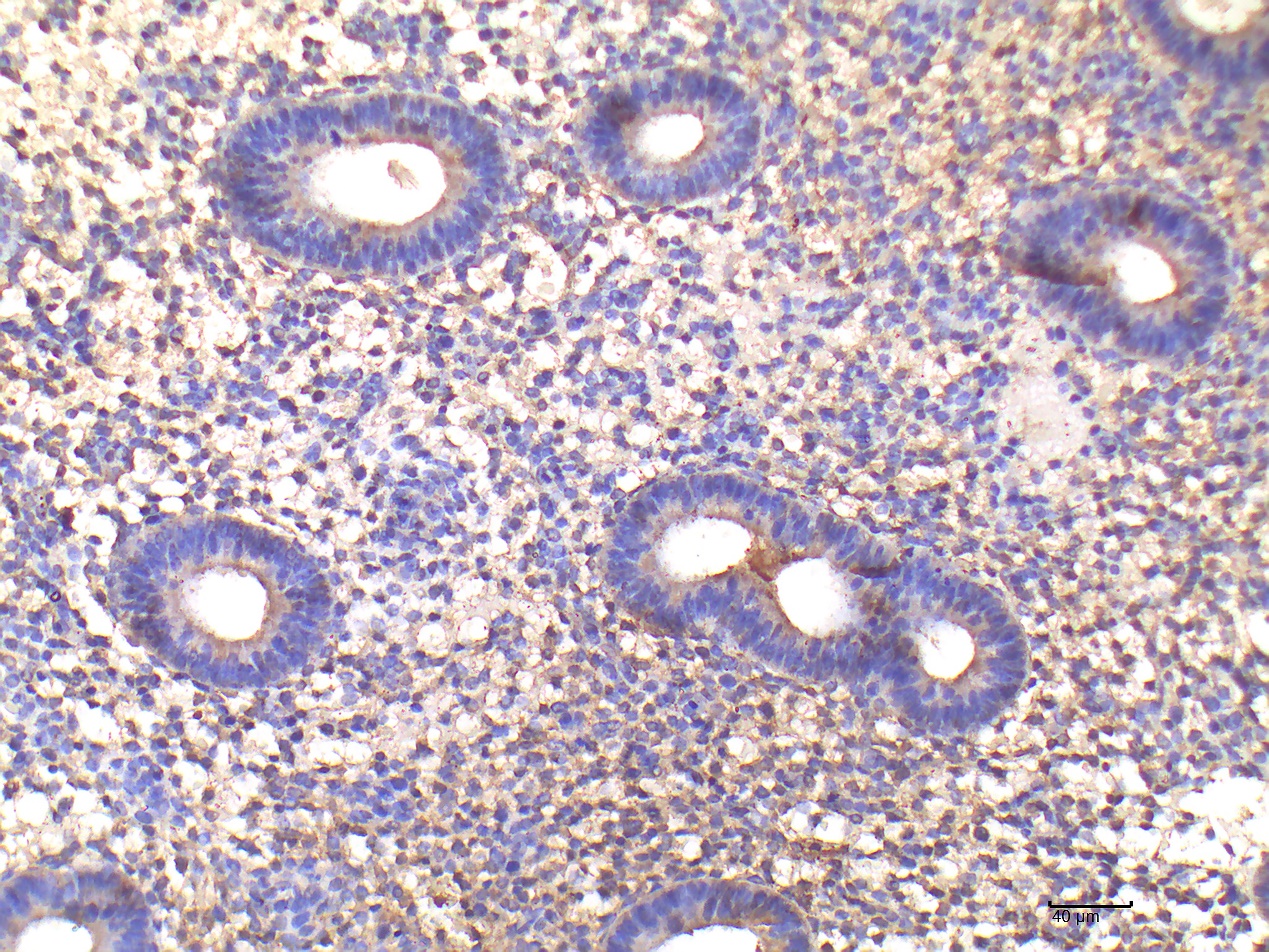


**MLH1**

**MSH2**

**MSH6**

**MMR-D**

**(Case 2)**

**p53abn**

**(Case 8)**


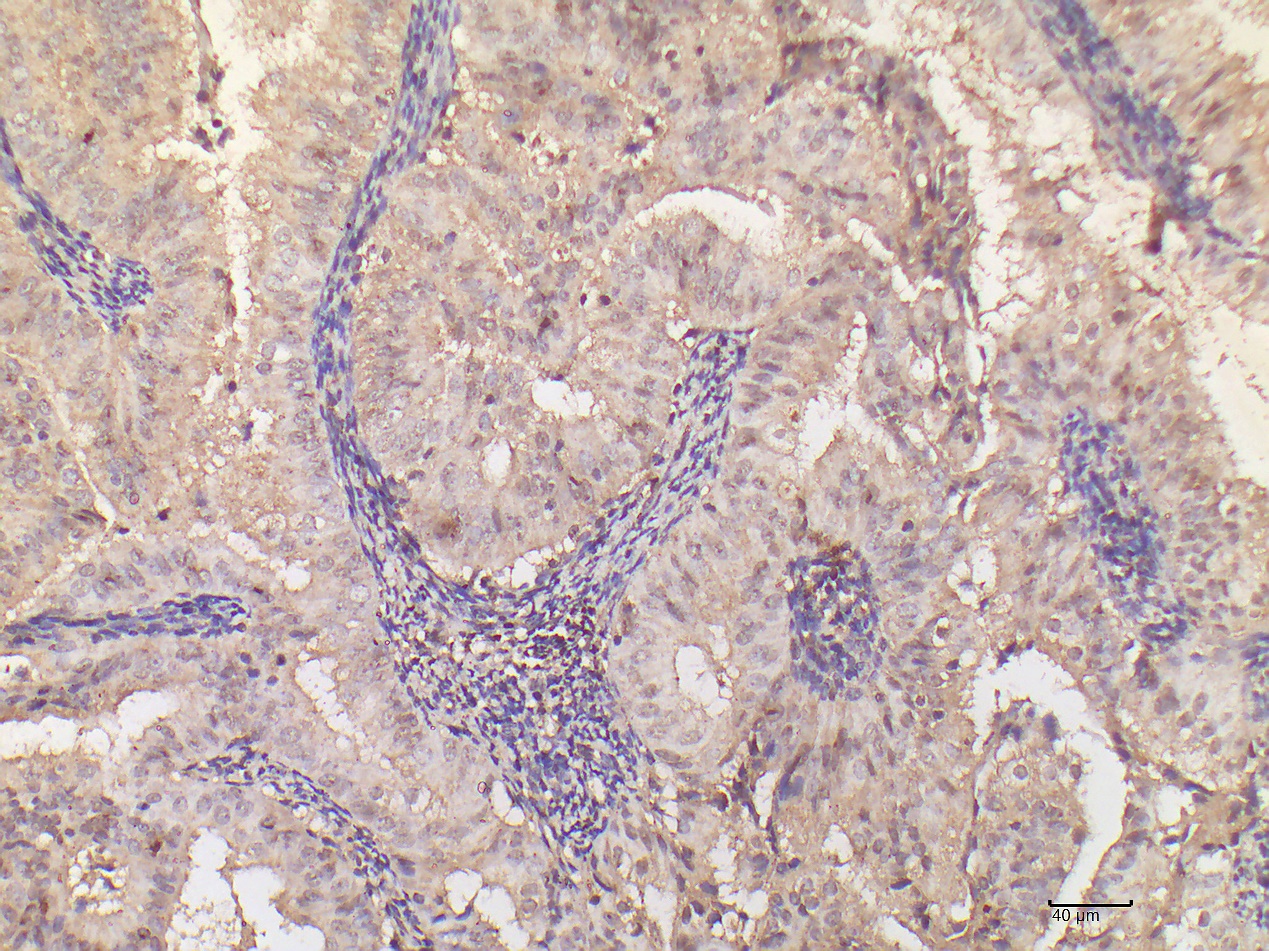

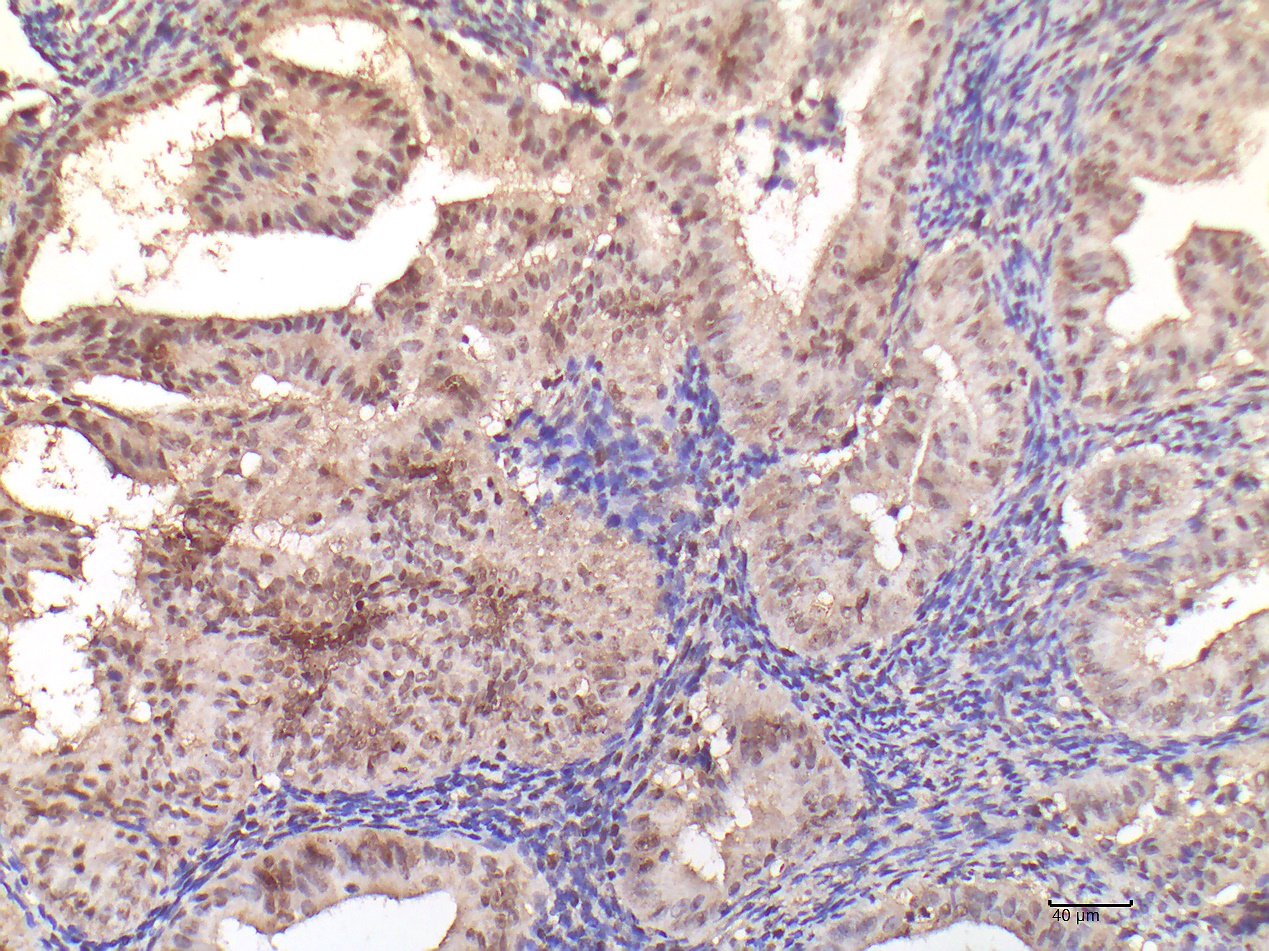

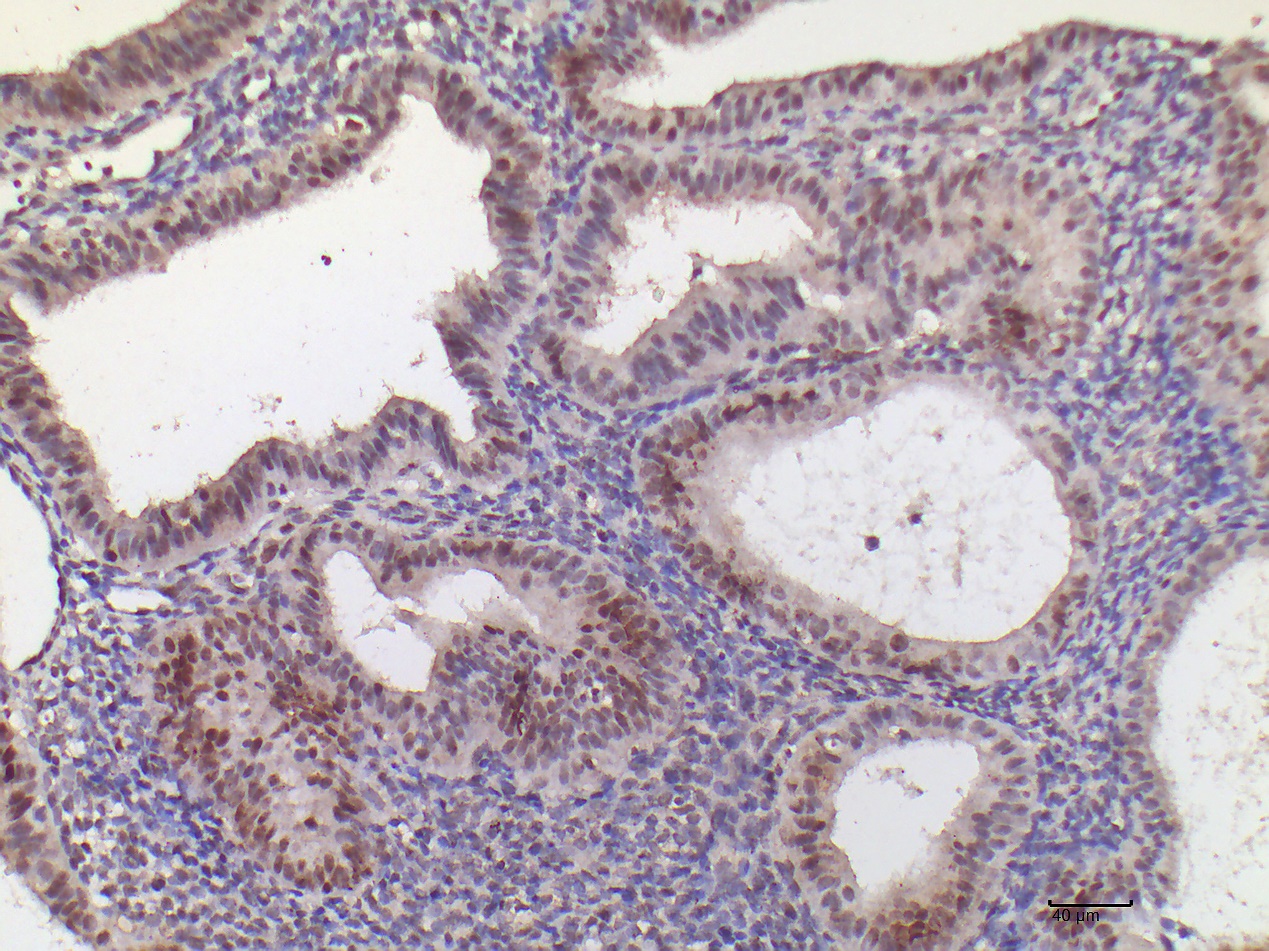

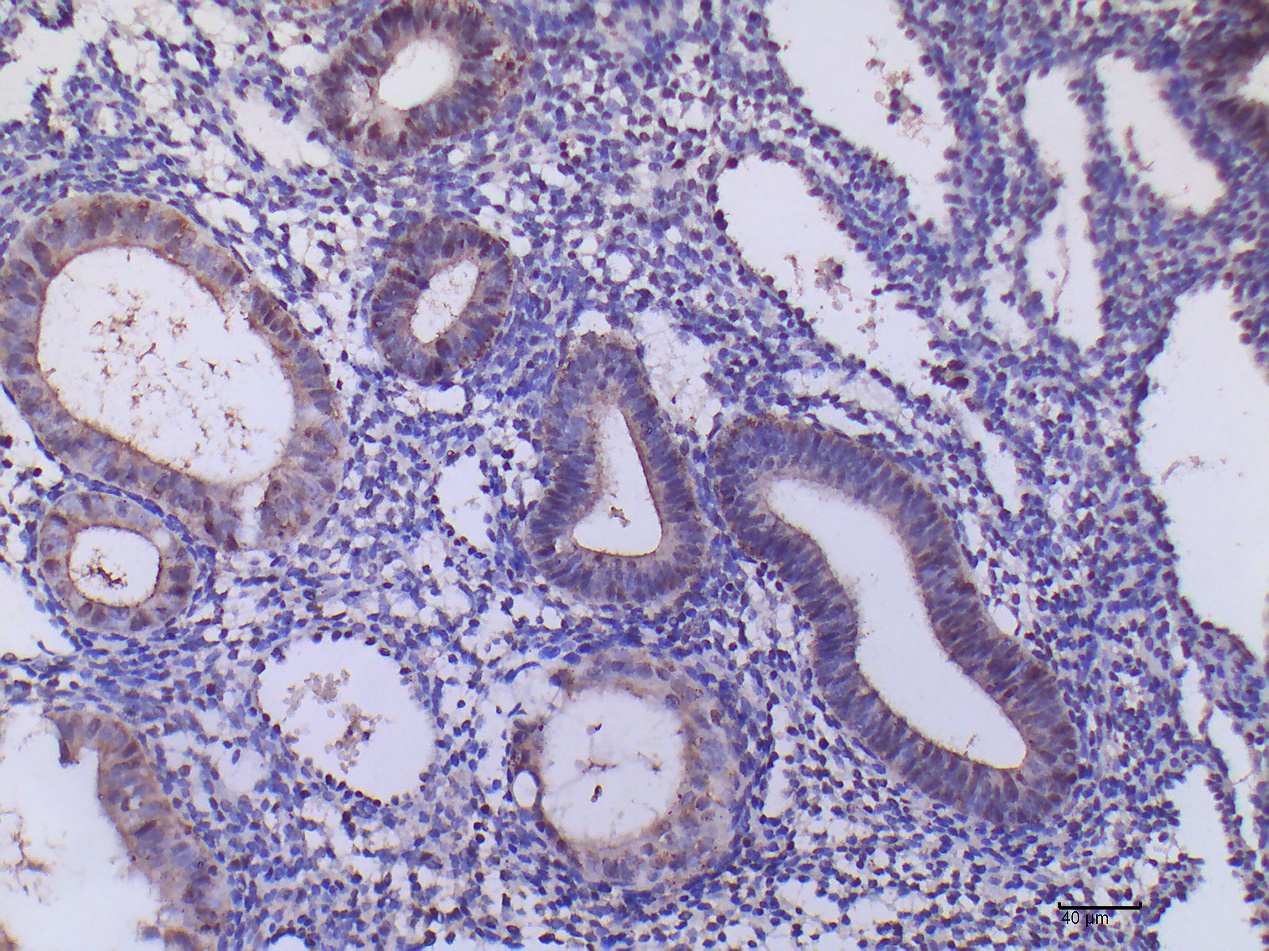

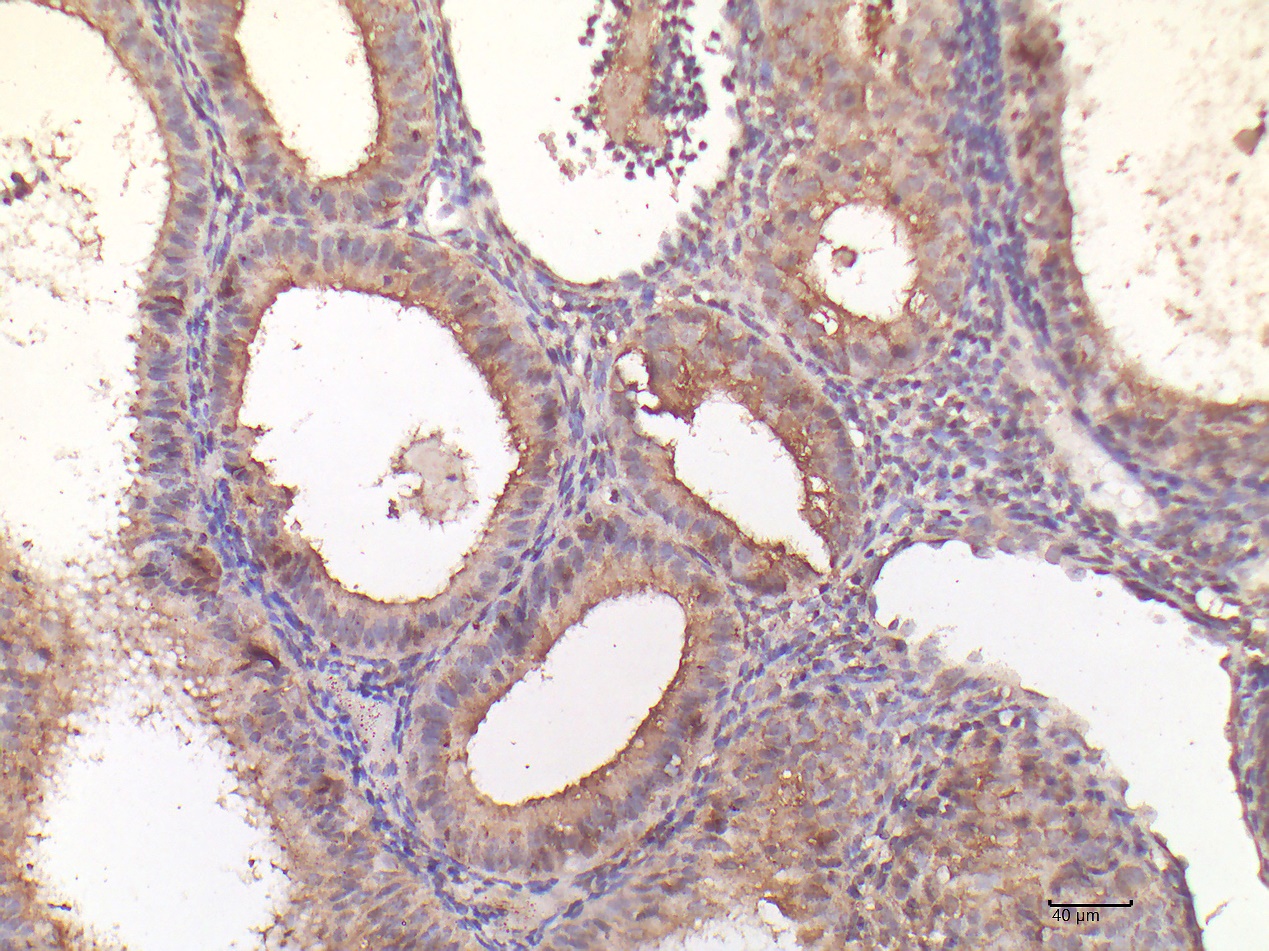


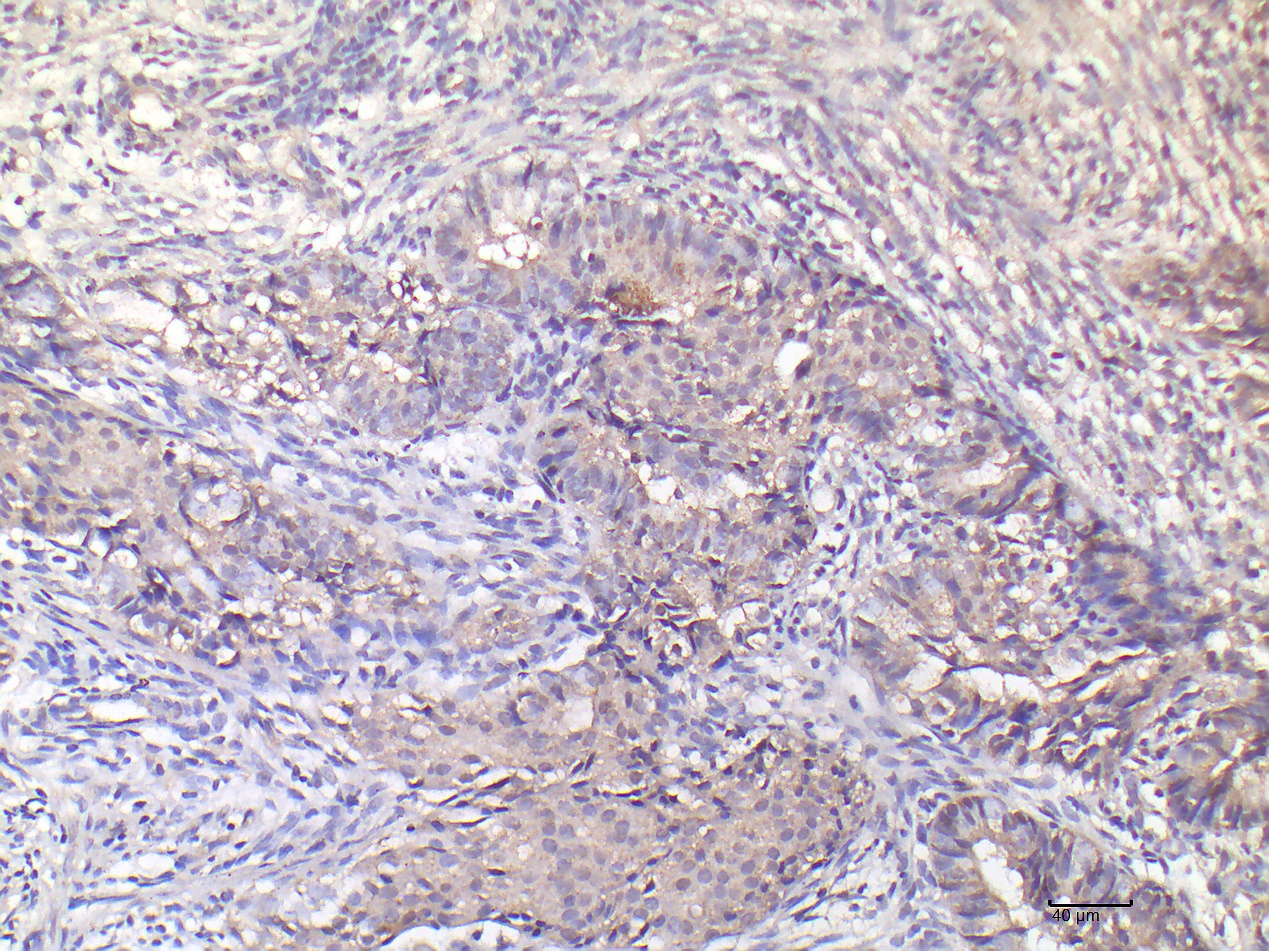

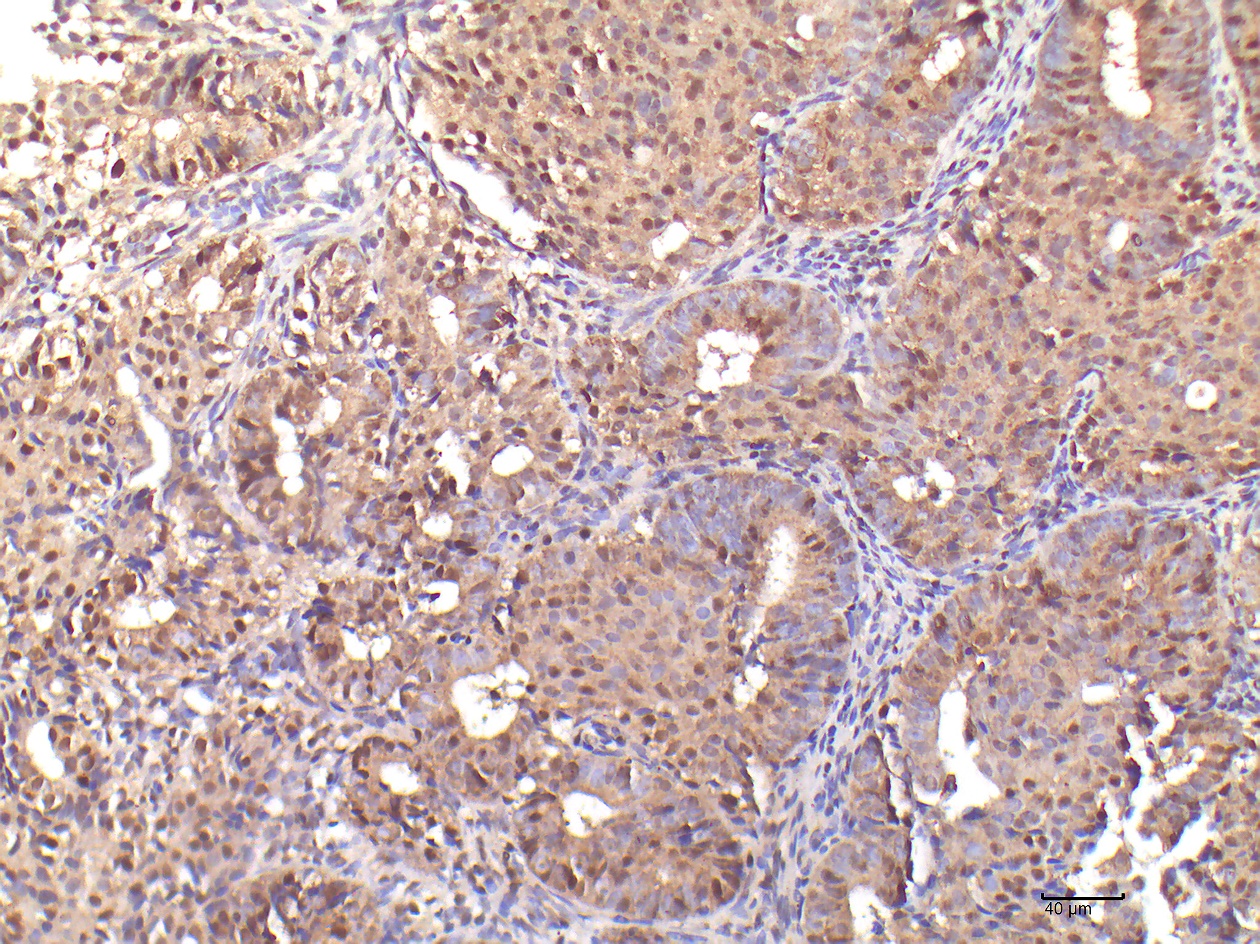

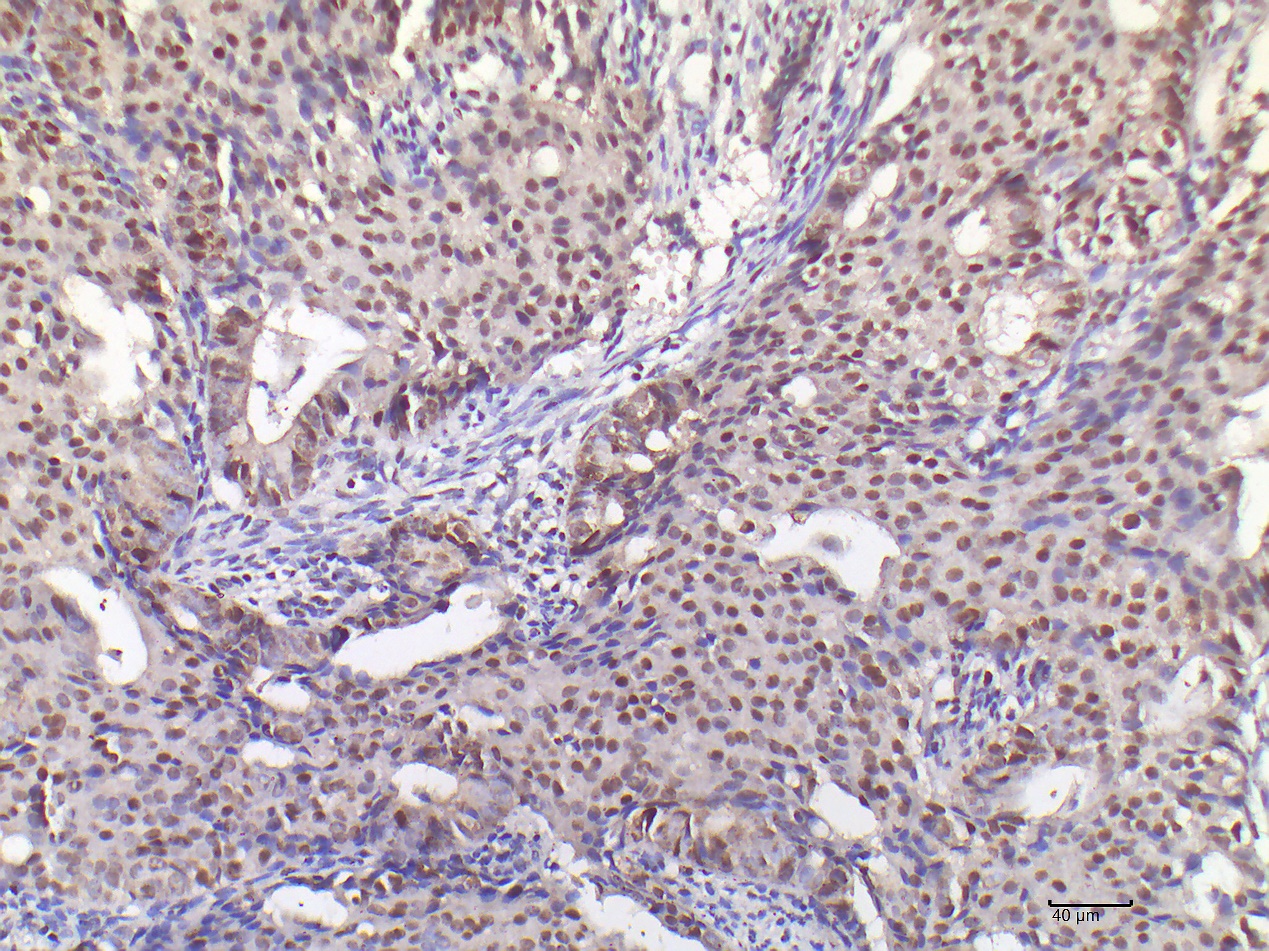

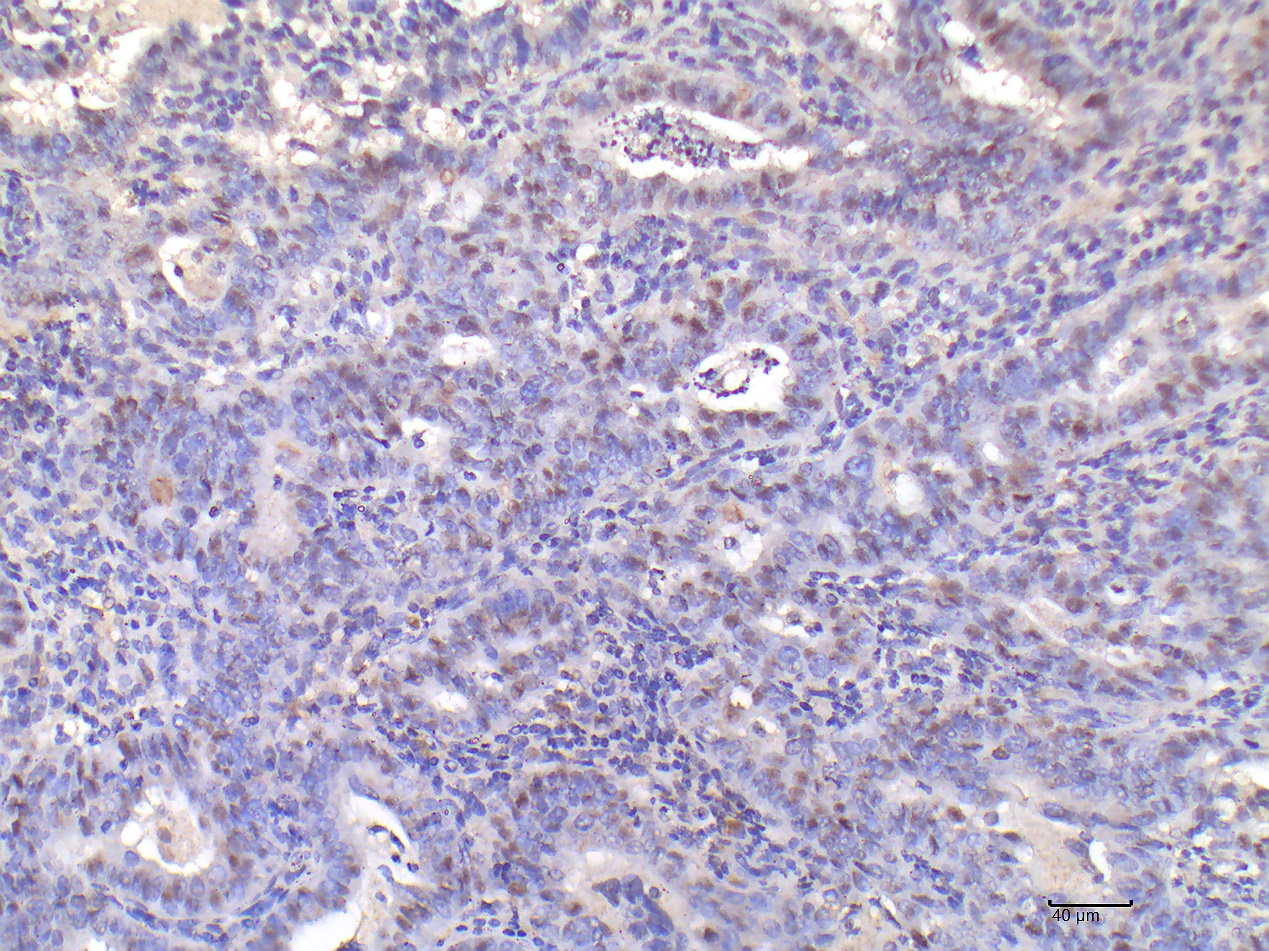

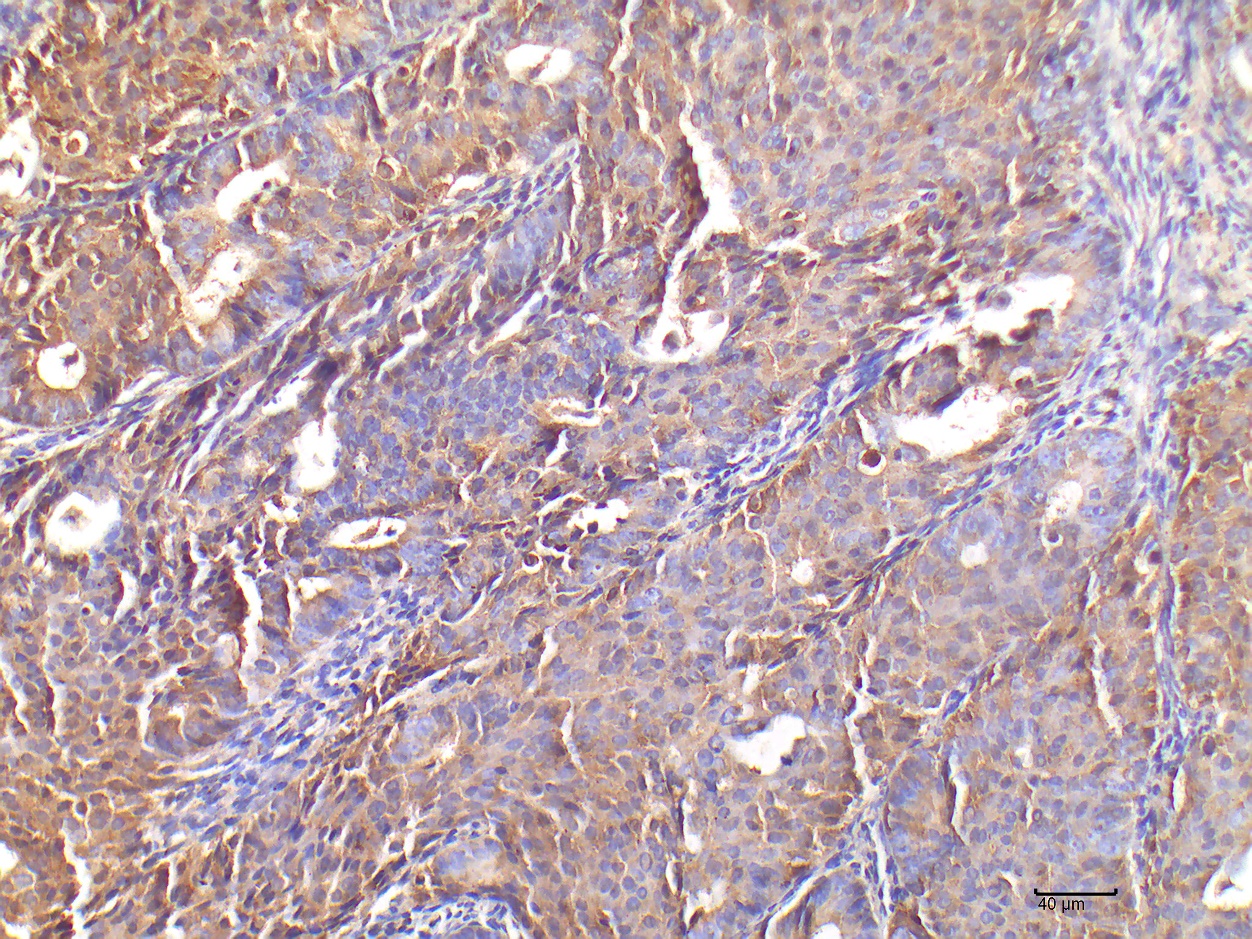


**MMR-D**

**(Case 12)**


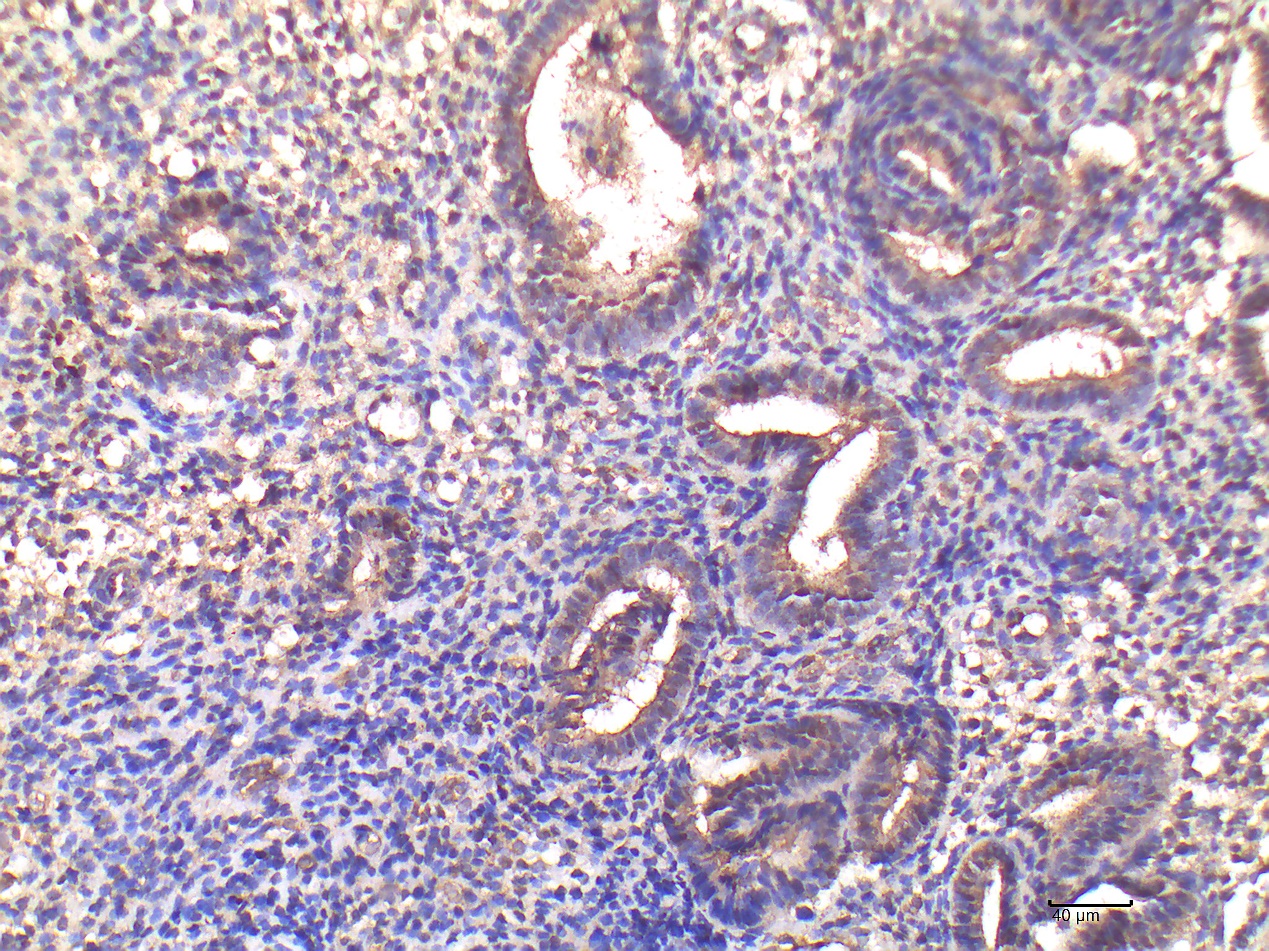

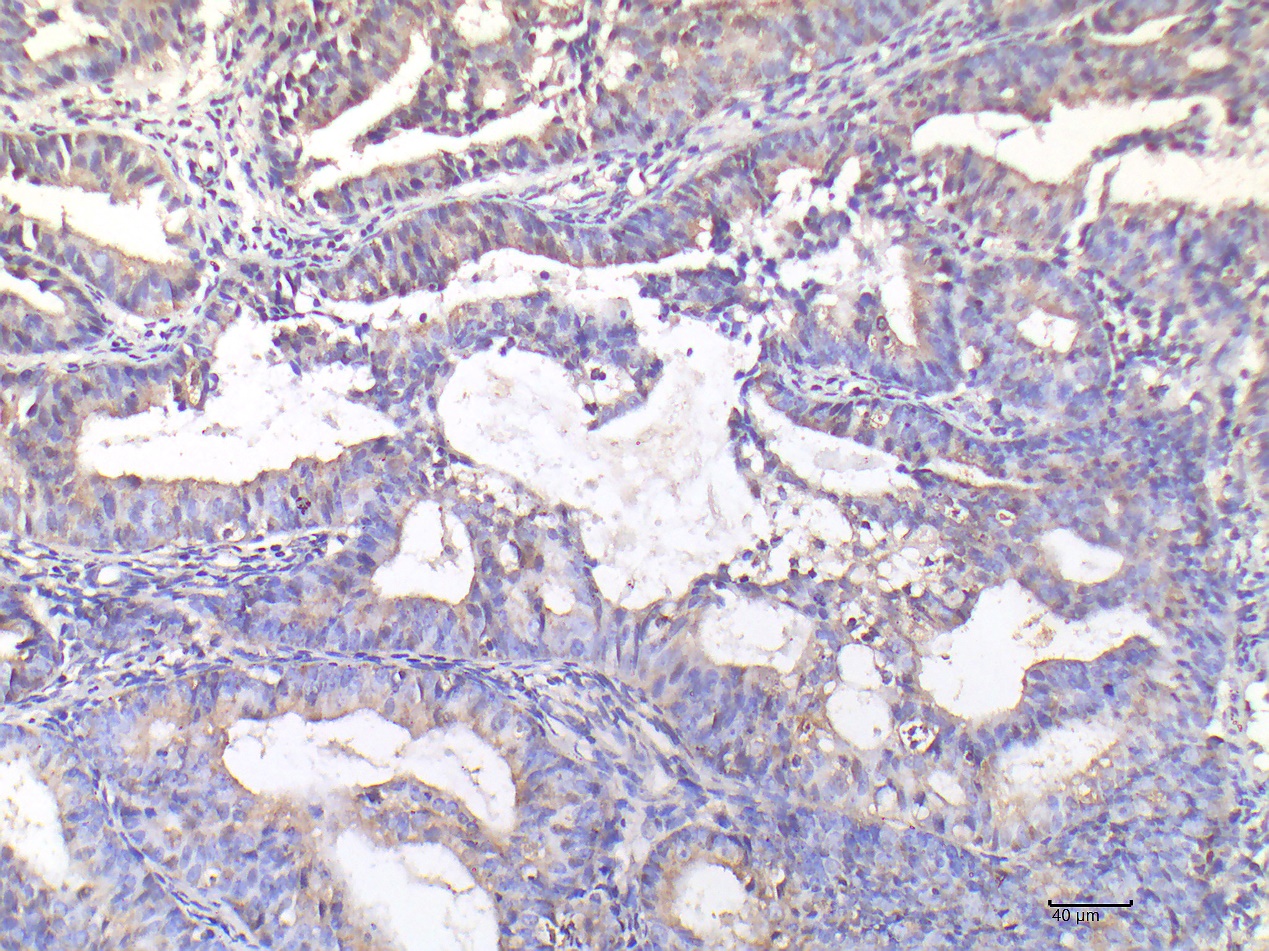

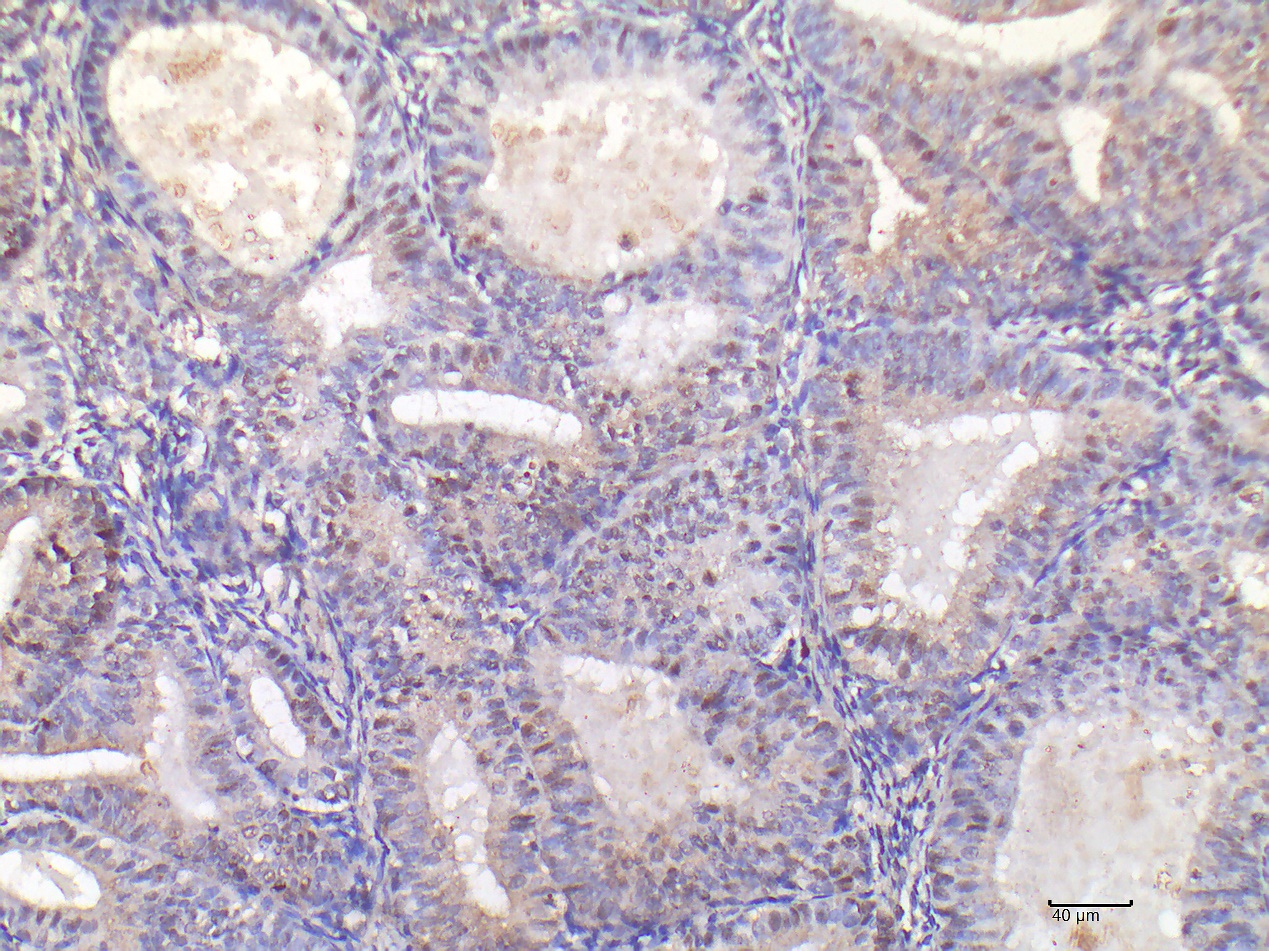

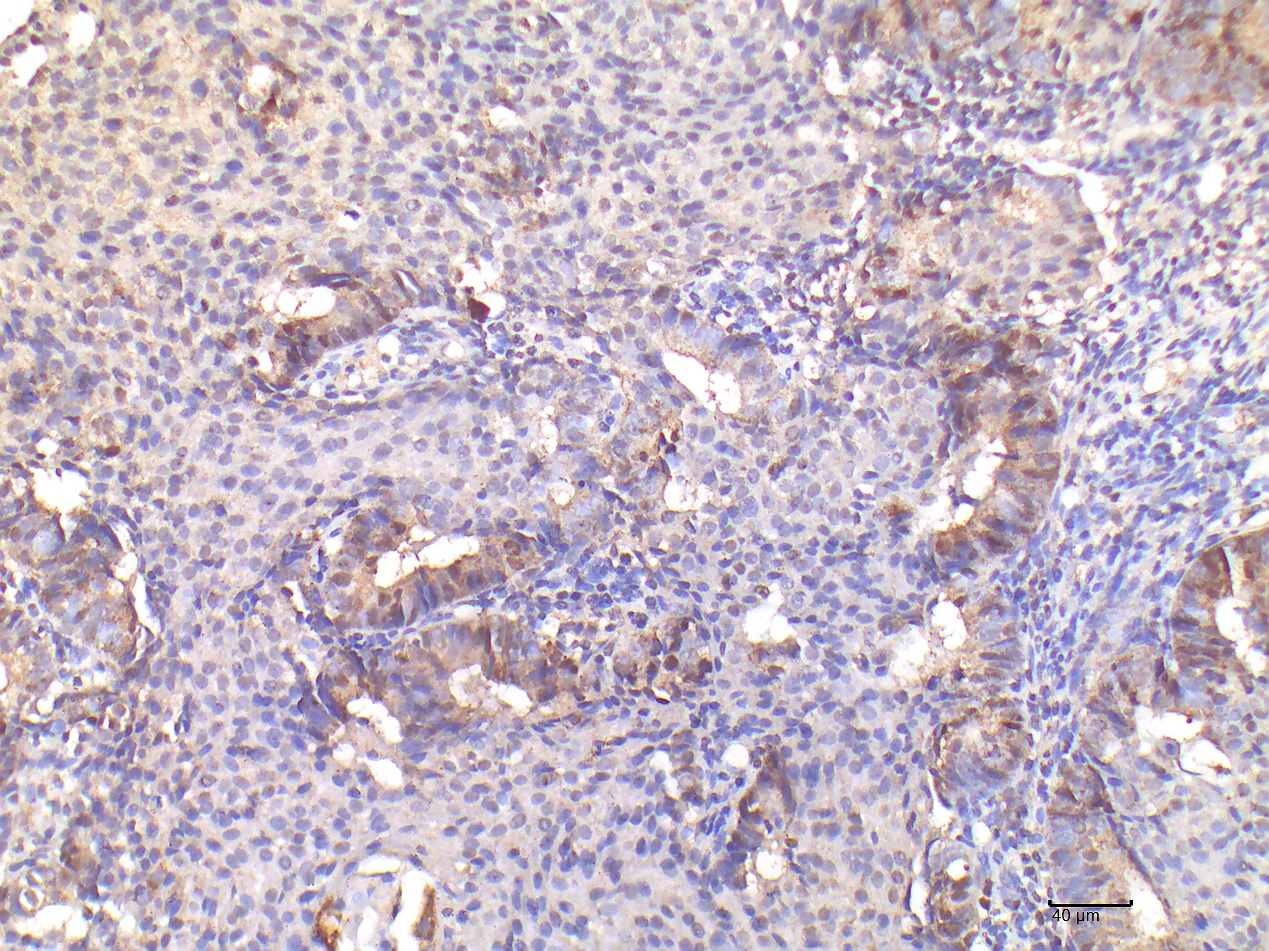

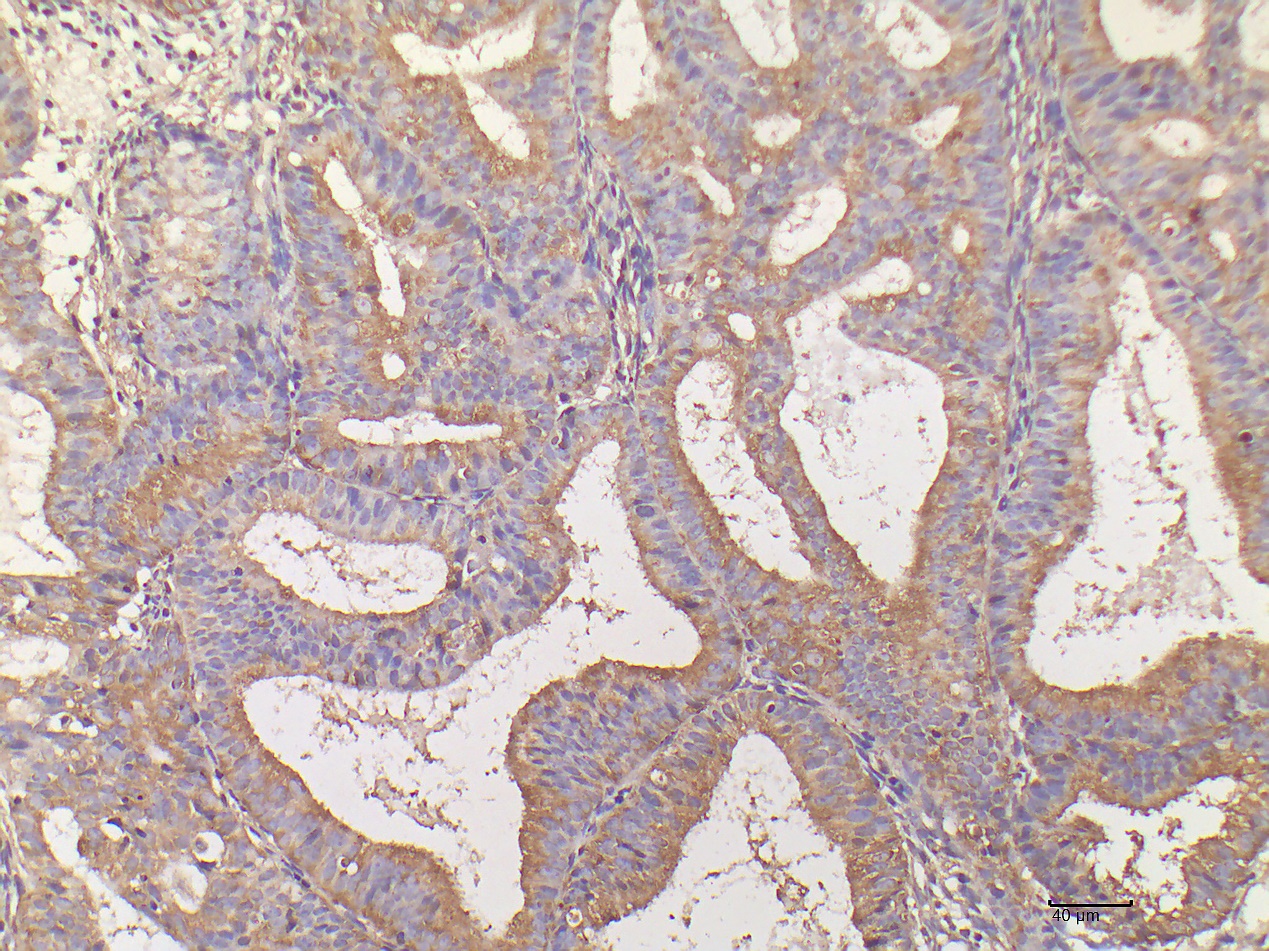


**MMR-D**

**(Case 13)**

**Supplementary Figure 1.** IHC results of p53, and mismatch repair protein (MLH1, MSH2, MSH6, and PMS2) staining of a case with a TP53 mutation (case 8), and mismatch repair deficiency (case 2, 12, 13). Case 2, 12, 13 was consistent with a mismatch repair deficiency, showing loss of MSH6, MSH6, MSH2 and MSH6 expression, respectively. Case 8 represents a tumor with a pathogenic p53 mutation and intact expression of mismatch repair protein (20×). Abbreviations: MMR-D, mismatch repair deficient.

eTable 1. Results of Quality Assessment of the 3 studies included in the analysis of outcomes of endometrial cancer in patients underwent FPT.

| Author, Year | Chung, 2020 | Falcone, 2019 | Puechl, 2021 |
| --- | --- | --- | --- |
| 1.Was the spectrum of patients representative of the patients who will receive the test in practice? | Yes | Yes | Yes |
| 2.Were selection criteria clearly described? | Yes | Yes | Yes |
| 3.Ascertainment of exposure | Secure record | Secure record | Secure record |
| 4.Is the diagnostic test likely to correctly classify the target condition? | Yes | Yes | Yes |
| 5.Type of diagnostic test | D&C,  hysteroscopy | Hysteroscopy | D&C,  hysteroscopy |
| 6.Was there an additional clinical test conducted before the diagnostic test? | No | No | No |
| 7.Did patients receive the same diagnostic test regardless of the additional test result? | N/A | N/A | N/A |
| 8.Was the diagnostic test independent of the additional test? | N/A | N/A | N/A |
| 9.Were the same clinical data available when test results were interpreted as would be available when the test is used in practice? | Yes | Yes | Yes |

eTable 2. Results of risk of bias assessment of the 3 studies included in the analysis of outcomes of endometrial cancer in patients underwent FPT

| **Quality in prognostic studies (QUIPS)** | | | | | | |
| --- | --- | --- | --- | --- | --- | --- |
|  | *Chung et al.*  *(2020)* | *Falcone*  *et al. (2019)* | | *Puechl et al.*  *(2021)* | | |
| **Study participation** | | | | | | |
| Adequate participation | n.a. | | n.a | | n.a. | |
| Source of population | 🗸 | | 🗸 | | 🗸 | |
| Baseline study sample | 🗸 | | 🗸 | | 🗸 | |
| Sampling frame & recruitment | 🗸 | | 🗸 | | 🗸 | |
| Period & place of recruitment | 🗸 | | 🗸 | | 🗸 | |
| Inclusion & Exclusion criteria | 🗸 | | 🗸 | | 🗸 | |
| Assessment of bias | Low bias | | Low bias | | Low bias | |
| **Study Attrition** | | | | | | |
| Response rate | 🗸 | | 🗸 | | 🗸 | |
| Information about participants who dropped out | 🗸 | | 🗸 | | 🗸 | |
| Reasons for loss-to-follow-up | 🗸 | | 🗸 | | 🗸 | |
| Description of participants lost to follow-up | 🗸 | | 🗸 | | 🗸 | |
| Differences between participants | 🗸 | | 🗸 | | x | |
| Assessment of bias | Low bias | | Low bias | | Low bias | |
| **Prognostic factor measurement** | | | | | | |
| Definition of PF | 🗸 | | 🗸 | | 🗸 | |
| Valid and reliable measurement of PF | 🗸 | | 🗸 | | 🗸 | |
| Continuous variables/cut points | 🗸 | | 🗸 | | 🗸 | |
| Consistency of method and setting of measurement | 🗸 | | 🗸 | | 🗸 | |
| Proportion of data available for analysis | 🗸 | | 🗸 | | 🗸 | |
| Method used for missing data | ? | | ? | | ? | |
| Assessment of bias | Low bias | | Low bias | | Low bias | |
| **Outcome measurement** | | | | | | |
| Definition of the outcome | 🗸 | | 🗸 | | / | |
| Valid and reliable measurement of outcome | 🗸 | | 🗸 | | 🗸 | |
| Consistency of method and setting of measurement | 🗸 | | 🗸 | | 🗸 | |
| Assessment of bias | Low bias | | Low bias | | Low bias | |
| **Study Confounding** | | | | | | |
| Important confounders measured* | 🗸 | | 🗸 | | 🗸 | |
| Clear definitions of important confounders | / | | / | | / | |
| Valid and reliable measurement of confounders | 🗸 | | 🗸 | | 🗸 | |
| Consistency of method and setting of measurement | 🗸 | | 🗸 | | 🗸 | |
| Appropriate methods for missing data | ? | | / | | ? | |
| Important confounders are accounted for in study design | 🗸 | | 🗸 | | 🗸 | |
| Important confounders are accounted for in analysis | 🗸 | | 🗸 | | 🗸 | |
| Assessment of bias | Low bias | | Low bias | | Low bias | |
| **Statistical analysis and reporting** | | | | | | |
| Sufficient presentation of data to assess adequacy of analytic strategy | 🗸 | | 🗸 | | 🗸 | |
| Adequate strategy for model building | n.a. | | n.a. | | n.a. | |
| Adequate statistical model | 🗸 | | 🗸 | | 🗸 | |
| No selective reporting | 🗸 | | 🗸 | | 🗸 | |
| Assessment of bias | Low bias | | Low bias | | Low bias | |
| Total score | Low bias | Low bias | | | | Low bias |

*important confounders: age, BMI, FIGO stage, tumor grade

🗸 fulfilled

/ partially fulfilled

X not fulfilled

? unknown

n.a. not applicable
